# Prospective Longitudinal Associations Between Adverse Childhood Experiences and Adult Mental Health Outcomes: Systematic Review and Meta-Analysis

**DOI:** 10.1177/15248380251358223

**Published:** 2025-08-17

**Authors:** Christina Thurston, Aja Louise Murray, Hannabeth Franchino-Olsen, Mpho Silima, Chad Lance Hemady, Franziska Meinck

**Affiliations:** 1 University of Edinburgh, UK; 2 The Ohio State University, Columbus, OH, USA; 3 University of the Witwatersrand, Johannesburg, South Africa; 4 Youth Endowment Fund, London, UK; 5 North-West University, Potchefstroom, South Africa

**Keywords:** mental health and violence, child abuse, violence exposure, neglect, child abuse, bullying

## Abstract

Research suggests a strong, dose–response relationship between adverse childhood experiences (ACEs) and poor adult mental health outcomes. This systematic review and meta-analysis aimed to systematically investigate the existence and strength of prospective associations between ACEs and adult mental health outcomes: anxiety, depression, psychotic-like experiences, post-traumatic stress disorder (PTSD), self-harm, and suicidality. We searched 12 electronic databases for publications after 1990. A narrative synthesis of included studies and random-effects meta-analyses with moderation were completed for all outcomes, excluding self-harm. In total, 62 studies from 15 countries were included. Most studies were from the United States; 95% of publications (*N* = 59) came from high-income countries (HICs) and 5% (*N* = 3) from upper-middle-income countries. Pooled associations between ACEs and adult mental illness were strongest for PTSD (OR = 2.26; 95% CI [1.75, 2.77]), followed by anxiety (OR = 1.78; 95% CI [1.45, 2.11]), depression (OR = 1.61; 95% CI [1.45, 1.76]), psychotic-like experiences (OR = 1.34; 95% CI [1.13, 1.54]), and suicidality (OR = 1.28; 95% CI [1.13, 1.43]). Moderation analyses suggested ACEs with a violence or maltreatment component were significant risk factors for adult depression compared to household ACEs, and that study location was a significant moderator in the depression, anxiety, and PTSD models. Further moderation effects will be discussed. Findings confirm ACEs are a significant risk factor for mental ill-health in adulthood. Our review highlights the urgent need for research exploring associations between ACEs measured in childhood and adult mental illness outside of HIC settings.

## Background

Adverse childhood experiences (ACEs) can broadly be defined as potentially traumatic life events occurring in the first 18 years of life (Centers for Disease Control and Prevention, 2019). These experiences encompass a spectrum of adversities that can be categorized into three overarching classifications: child abuse (emotional, sexual, and physical), child neglect (emotional and physical), and household dysfunction (alcohol and/or drug abuse in the house, imprisoned family member, mother treated violently, and parental loss, separation, or divorce) (Burke et al., 2011). While these ACEs remain the most heavily researched, additional ACEs are recognized, such as being bullied (van Dam et al., 2012), community and collective violence (El Mhamdi et al., 2017), parental mortality and morbidity (Mittendorfer-Rutz et al., 2012), child marriage (Le Strat et al., 2011), and child trafficking (Ottisova et al., 2018).

Over the last few decades, extensive research has explored the relationship between ACEs and subsequent poorer cognitive, emotional, and behavioral outcomes (Felitti et al., 1998). Robust cross-sectional and longitudinal evidence indicates a heightened risk of developing psychiatric problems after exposure to ACEs, including depression (Chapman et al., 2004), anxiety disorders (Poole et al., 2017), suicidal ideation (Stansfeld et al., 2017), and psychosis (Karcher et al., 2020). The increasing body of extant literature underscores ACEs as a significant public health problem (Sacks & Murphy, 2018), and emerging research has recognized adult mental illness as one of the largest public financial burdens associated with ACEs (Hughes et al., 2020).

In recent years, ACE studies have also been subject to multiple systematic reviews and meta-analyses. In their systematic review and meta-analysis, Hughes et al. (2017) demonstrated the significant, deleterious effects of multiple ACEs (four or more) on lifelong health. Other systematic reviews include Norman et al. (2012) and Kalmakis and Chandler (2015), whose results suggested significant associations between ACEs and various long-term mental health outcomes and health-harming behaviors, including depressive disorders, suicide attempts, PTSD, substance misuse, and sexual risk behavior. Sahle et al.’ (2022) recent umbrella review also confirmed strong, significant associations between ACEs and common mental disorders, and a review by Bellis et al. (2019) highlighted that ACEs remain a preventable contributor to some of the most significant public health challenges and financial burdens on healthcare systems across Europe and North America.

While several prior reviews have established associations between ACEs and mental health outcomes, they have largely relied on retrospectively measured ACEs, limiting temporal inference and increasing the risk of bias (Sahle et al., 2022). The rationale for this review stems from growing concerns about the validity of retrospective ACEs reporting, which dominates the literature but is vulnerable to recall bias, memory decay, and mood-congruent distortion—particularly among individuals with mental health conditions (Baldwin et al., 2019; Reuben et al., 2016). Empirical comparisons between prospective and retrospective reports show poor agreement (Newbury et al., 2018), with the reporting methods capturing two distinct groups of individuals. To address this, we include only prospective, longitudinal studies in which ACEs were measured in childhood and mental health outcomes assessed in adulthood, in line with recent recommendations (Baldwin et al., 2019; Lacey & Minnis, 2020).

This review also extends prior work by: (a) including grey literature in the inclusion criteria to reduce publication bias and support broader global representation, particularly from regions beyond high-income countries (HICs), even though low- and middle-income country (LMIC) studies remain underrepresented in this evidence base; (b) expanding the scope of outcomes beyond psychiatric diagnoses to include subclinical symptoms; and (c) employing a more comprehensive, updated search strategy. These choices aim to provide a more temporally robust and globally inclusive synthesis of ACE–mental health associations.

### Aim and Review Questions

The main aim of this systematic review and meta-analysis was to address the gap in the literature by exploring the prospective associations between ACEs and the specific adult mental ill-health outcomes of depression, anxiety, PTSD, psychotic-like experiences, suicidality, and self-harm in prospective longitudinal research globally. We addressed the following questions:

1. What are the associations between ACEs and depression, anxiety, PTSD, suicidal ideation, self-harm, and psychotic-like experiences in adulthood with a specific interest in the prevalence of research conducted in HICs versus LMICs?
2. Which ACEs have the largest negative associations with adult mental health?
3. Which geographical locations does the evidence stem from, and are the associations between ACEs and adult mental ill-health moderated by the geographical location of the study?
4. Is the association between ACEs and adult mental ill-health moderated by study design or analysis?
5. What is the quality of studies looking at longitudinal associations between ACEs and mental health outcomes?

We combined questions 2 and 4 of our protocol (Thurston et al., 2023), which now appear as a single question 3. Certain questions outlined in our protocol (Thurston et al., 2023) were not answered as they were contingent on enough studies fitting the criteria. The questions that could not be answered asked whether there would be a cumulative effect of ACEs on mental health outcomes, and whether there were moderating effects of age of onset of first adversity and peer-reviewed status on the association between ACEs and mental health outcomes. This was due to either lack of data included in studies, or excessive heterogeneity in ACE conceptualization, measurement, and scoring.

## Methods

### Inclusion and Exclusion Criteria

We adopted the Population-Exposure-Outcome model to aid in outlining the inclusion and exclusion criteria seen in Supplemental Appendices A and B, respectively.

### Information Sources

Twelve electronic databases were searched: Embase, PsycINFO, MEDLINE (Ovid version), and Global Health through the Ovid interface. ProQuest was used to search Public Affairs Information Service, Dissertations and Theses, Sociology Database (including Sociological Abstracts and Social Services Abstracts), PTSDpubs (formerly PILOTS) and Applied Social Sciences Index and Abstracts (ASSIA). CINAHL, WHO Global Index Medicus, and WHO Violence Info were also searched. The search was first conducted throughout June 2021. However, delays in protocol publication resulted in authors rerunning the same search in March 2023 to capture more up to date research for this current manuscript. This update of search timeframe was stated in the protocol (Thurston et al., 2023). The searches were limited to publication date from 1990 onward and to human subjects in databases that included this limiter. This specific period of 1990 onward was chosen as it aligned with the drafting of the United Nations Convention on the Rights of the Child (UNCRC) by the United Nations (UNCRC, 2019). It should be noted that studies published after 1990 that used data from cohorts prior to 1990 were still eligible if all inclusion criteria were satisfied. This was decided as the study rationale, research design, research questions, analyses, and findings would be interpreted with knowledge from the UNCRC, including a universal definition of when childhood ends and detailed conceptualizations of child protection and maltreatment (Nadan et al., 2015). The English language specification was manually screened. To ensure literature saturation, the authors emailed authors of known large cohort studies in the relevant field of research to query whether they had any research that was unfinished/ in the process of being published. Search terms and a table of definitions of key concepts can be found in the appendices of our protocol (Thurston et al., 2023).

### Search Strategy

Examples of the search strategies can be found in the appendices of our protocol (Thurston et al., 2023) and in Supplemental Appendix C. The search strategy was altered to account for varying syntax, limiters, and expanders in different databases.

### Data Management

Studies identified by the database searches were exported to Zotero where they were de-duplicated. Then, references were transformed into RIS file format and exported to Covidence (a systematic review management software). Once imported to Covidence, duplicates were checked for and removed again.

### Selection and Collection Process

Abstracts and titles were independently double screened to determine whether the studies met the inclusion criteria. Next, the remaining papers were subjected to a full-text screen for the assessment of inclusion by two reviewers. Any discrepancies in the decision to include a study in the final review were resolved by team discussion or by a third independent reviewer.

**Figure 1.**
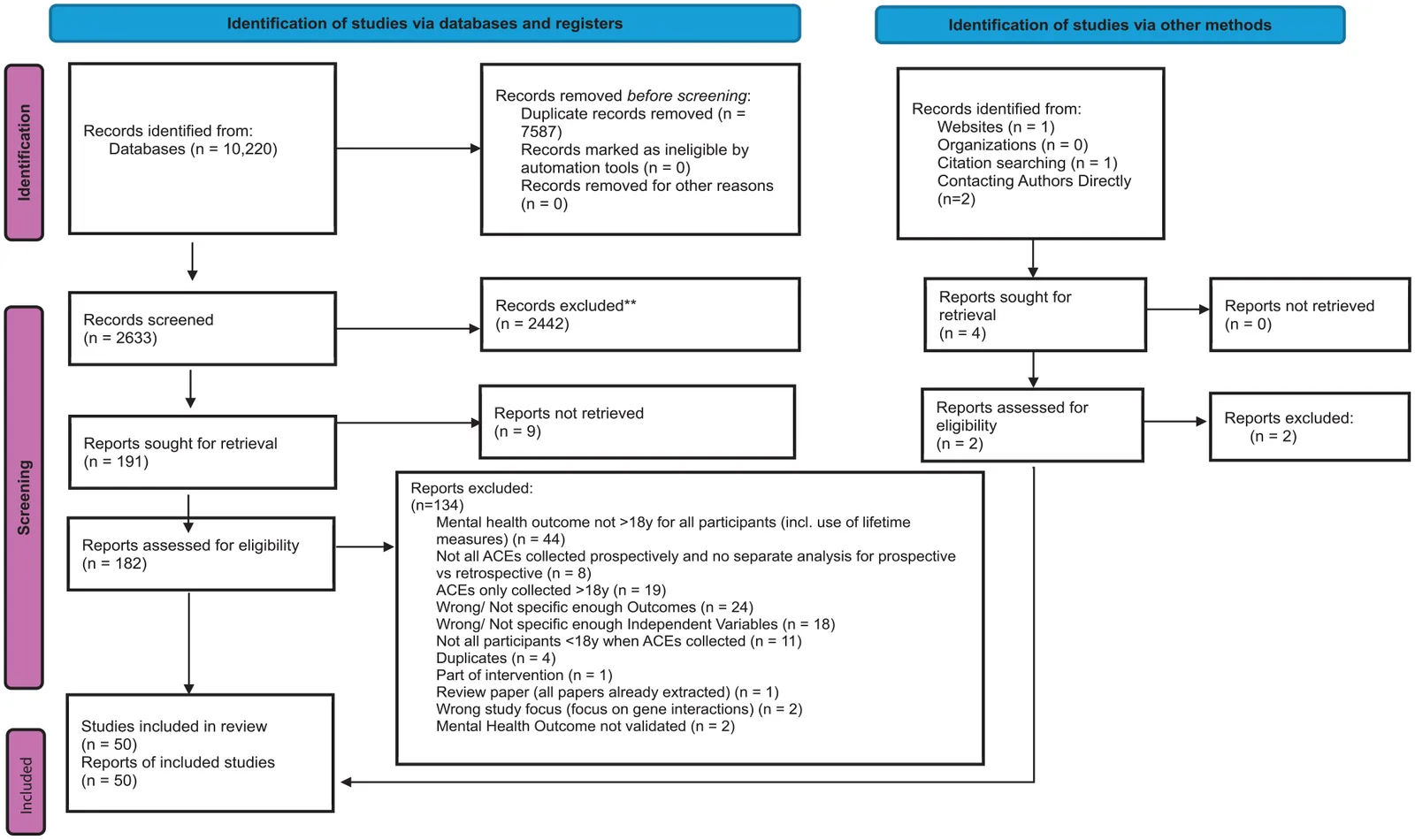
Flowchart of the screening process from the first search conducted for the period 1990 to May 2021. *Source*. Page et al. (2021). For more information, visit: http://www.prisma-statement.org/

**Figure 2.**
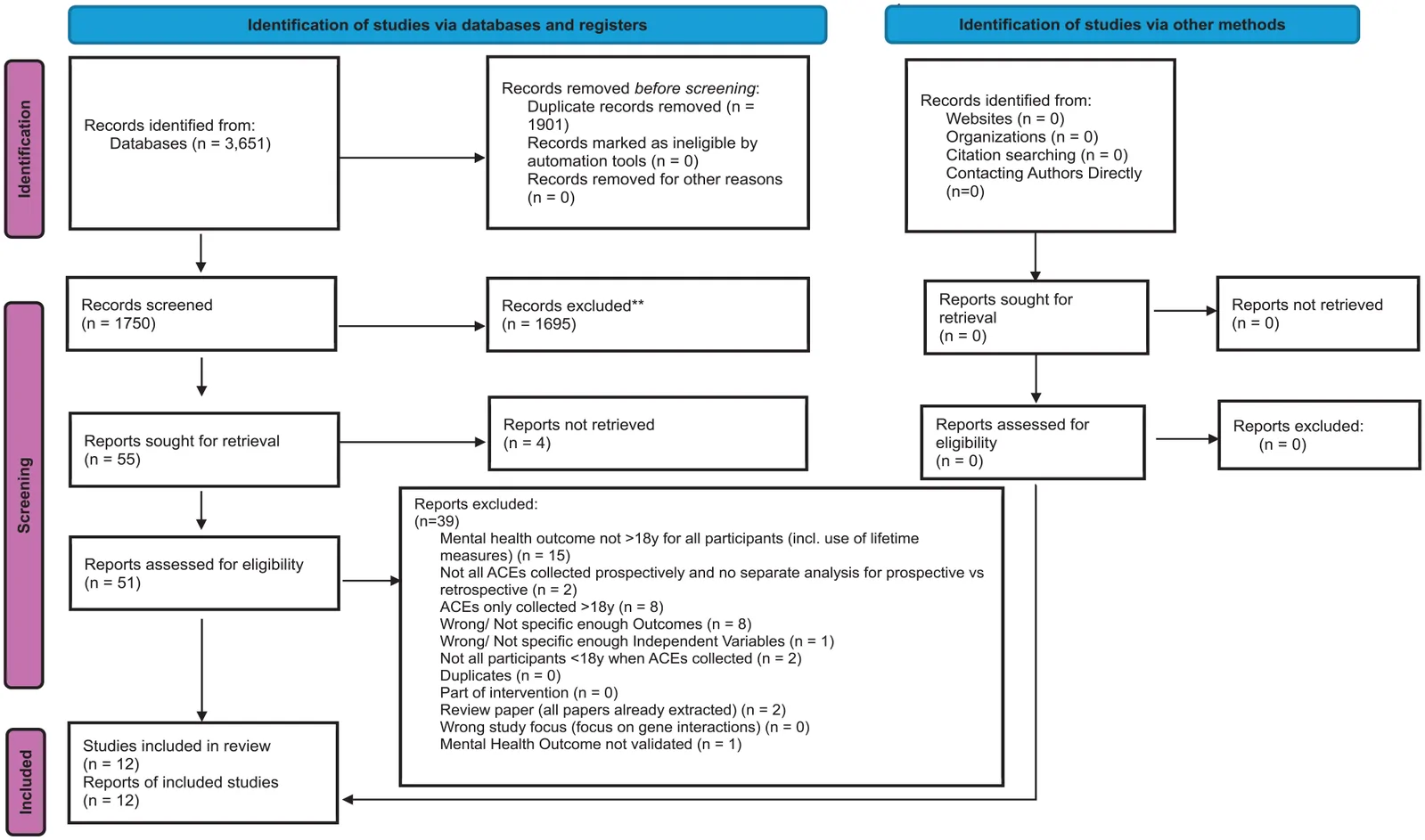
Flowchart of the screening process from the updated search results from June 2021 to March 2023. *Source*. Page et al. (2021). For more information, visit: http://www.prisma-statement.org/

After double-screening 4383 titles and abstracts derived from database searches, hand-searching the reference lists of any similar reviews, and author/expert recommendations, 244 studies remained eligible for full-text screening (see Figure 1 for the flowchart of the screening process from the first search conducted in 2021 for the period 1990 to May 2021. See Figure 2 for the flowchart of the screening process from the updated search results from June 2021 to March 2023). Sixty-two studies met all the eligibility criteria and were suitable for data extraction. Supplemental Appendix D provides a full reference list of these studies.

The final data extracted from the remaining studies were stored in a spreadsheet on Covidence. The data extracted by reviewers included general information (first author, year of study, format e.g., article); more specific characteristics (setting, sample size, sample source, study design); variable information (measurement/ tool(s) used to collect ACE and mental health data, type of ACEs measured, source of ACEs reporting, type of mental health outcomes measured, age adversity/ mental health was recorded); and analysis (metrics, adjustments, results).

Of the 62 studies included for narrative synthesis, the majority were conducted in the USA (*N* = 20) and the rest were from 13 other countries and 1 Europe-wide study (see Supplemental Appendix E for study summary characteristics). There was a notable unequal spread of studies across different country income levels. Most studies were conducted in HICs (*N* = 59) and significantly fewer were conducted in LMICs (*N* = 3). Study years ranged from 1996 to 2023 and sample sizes ranged from 151 (Llabre & Hadi, 2009) to 487,141 (Björkenstam et al., 2017).

While our protocol stated that for each study, we would extract and report sociodemographic factors (e.g., gender, socioeconomic status, ethnicity) and extract the prevalence of ACEs and mental health conditions, not enough studies included this information in an accessible and comparable format for our review to synthesize this information. While most studies provided this information, it was not disaggregated clearly (e.g., mental health outcome prevalence may have been split by gender and ethnicity in one study and not split by sociodemographic factors in another).

### Study Risk of Bias Assessment

The Newcastle-Ottawa Scale (NOS) for cohort studies and case-control studies (Wells et al., 2000) was used to assess study quality. The NOS implements a star system and a maximum of 8 stars can be awarded (A maximum award of 1 star per item within the domains Selection and Exposure and a maximum award of 2 stars for the domain of Comparability) (Stang, 2010). Two reviewers independently assessed the methodological quality of the studies included and any discrepancies in agreement were resolved by a third reviewer. In the protocol (Thurston et al., 2023) we asserted, we would not give each included study an overall quality score or “total star rating.” This was in line with limitations of overall quality scores highlighted at the time in the Cochrane Handbook for Systematic Review of Interventions (Higgins et al., 2023), including a lack of uniformity of quality appraisals across different quality scales being largely attributable to differing conceptualizations of “quality.” However, we chose to include a table detailing the number of stars given to each study in Supplemental Appendix F, as to better directly reflect the quality of the research.

### Certainty Assessment

We did not perform a formal certainty of evidence assessment using Grading of Recommendations, Assessment, Development and Evaluations (GRADE) or a comparable framework. While GRADE is a widely accepted tool for assessing the confidence in effect estimates, its application is most appropriate in systematic reviews of interventional studies or those informing clinical practice guidelines. In contrast, our review synthesized evidence from a methodologically heterogeneous body of observational, prospective longitudinal studies, which are not ideally suited to the GRADE framework due to several key limitations.

First, there was substantial methodological and analytic heterogeneity across studies, including variations in how ACEs and mental health outcomes were conceptualized, measured, and reported. Second, most studies reported unadjusted effect sizes, which were extracted or converted to odds ratios (ORs) for comparability across studies. However, differences in covariate adjustment and analytic approaches across studies precluded meaningful comparison of adjusted estimates. Third, the nonstandardized definitions and measurements of ACEs, including the use of study-specific items and composite scores, limited the feasibility of applying GRADE’s domains (e.g., indirectness, inconsistency, imprecision) in a uniform and rigorous way. Lastly, the presence of multilevel modeling to account for effect size dependence and significant between-study heterogeneity (as indicated by prediction intervals and I² statistics) would reduce the validity of GRADE ratings without introducing subjectivity.

Given these constraints, we prioritized a transparent approach by thoroughly assessing risk of bias at the study level using the NOS, conducting meta-regression to examine potential sources of heterogeneity, and discussing the limitations of the evidence base in detail.

### Synthesis Methods

We completed a narrative synthesis of studies to answer review questions 1–3, which included tabular summaries of the included studies and a discussion of the relationships within and between the studies. Enough studies with adequate similarity in design were identified by the database searches for meta-analyses to be conducted using the “metafor” (Viechtbauer, 2015) package in R. Thirty-nine out of the 62 studies were meta-analyzed. Twenty-three studies were unable to be included in the meta-analysis due to the fact that data for the appropriate conversion to unadjusted odds ratio statistics were not included in the paper (or insufficient information for successful conversion), too few studies were included for the mental health outcome to be meta-analyzed (self-harm), or the studies only presented adjusted effect sizes and not unadjusted effect sizes.

The only mental health outcome where there were not sufficient studies identified (minimum needed = 2 studies) for a meta-analysis was self-harm. Many of the studies measured participant exposure to more than one childhood adversity and thus reported multiple effect sizes. Fit indices were used to confirm that three-step meta-analytic models were necessary to account for any effect size dependency within studies for the models where depression, anxiety, and suicidality were outcomes (van den Noortgate et al., 2015). The remaining indices for psychotic-like experiences and PTSD indicated that fitting three levels would lead to overparameterization and thus remained at the two-level model.

Random-effects models were chosen as we predicted that the effect sizes reported may vary as a function of exposure, the measurement tools used, and differences in the populations from which the samples were drawn. Specifically, unadjusted ORs were computed in the meta-analysis and when study findings were not presented using ORs, the Campbell Collaboration Meta-Analysis Effect Size Calculator (Wilson, n.d.) was used to support effect size conversion. Conversions of different reporting methods were also completed such as exponentiating unstandardized logistic regression coefficients to ORs. Supplemental Appendix G provides a list of all studies where conversions were made and reasons for lack of conversion where applicable, and a full breakdown of individual study summary characteristics is available at https://osf.io/tgkxe.

Using meta-regressions, we were able to assess for moderating effects of year of publication, ACE type, ACE count, location, ACE source of reporting, and study design. Rather than conducting subgroup analyses, we employed moderation within a three-level meta-analytic framework to account for the nested structure of effect sizes. Subgroup analyses can introduce information loss and inflated Type Ι error rates in multilevel data, whereas moderation allows for more efficient and statistically appropriate assessment of heterogeneity across study-level characteristics (Assink & Wibbelink, 2016; Viechtbauer, 2010). This approach was chosen to ensure robust handling of dependencies across effect sizes within studies.

There were not enough studies that explicitly gave the age of each adversity to explore the moderating effects of the age of adversity onset. The measurement of ACEs was particularly heterogeneous across studies, with many including custom items to measure ACEs. Consequently, moderator analyses on measurement type were not feasible and would lack statistical power and robustness. In addition, only one study was not published, so we could not assess the moderating effect of publication status.

The I^2^ statistic was used to assess statistical heterogeneity. However, it should be noted that some research suggests I^2^ can be biased in small meta-analyses (von Hippel, 2015). To supplement the I^2^ statistic and enhance interpretation of meta-analytic results, prediction intervals will be reported to explore the dispersion of estimates (Deeks et al., 2019).

### Publication Bias

We used funnel plots to graphically assess publication bias for each outcome in the meta-analysis (Sterne & Harbord, 2004). Literature recommends extreme caution using funnel plots in analyses where the number of studies is fewer than 10 due to issues of reliability and low power (Fagerland, 2015; Sedgwick, 2013) and this was acknowledged in our analysis. The standard Egger’s regression test further checked for publication bias in the two-level models, where effect sizes were regressed on standard errors (SEs; Egger et al., 1997). There is currently no direct function to conduct an Egger’s bias test in meta-analytic models with more than two levels (Assink & Wibbelink, 2016; Fernández-Castilla et al., 2021). In instances with multiple effect sizes within studies, a number of extensions to the original Egger’s regression test have been suggested (Egger et al., 1997; Fernández-Castilla et al., 2021; Stanley & Doucouliagos, 2014), including the addition of moderators such as a measure of precision of the estimates into the multilevel meta-analytic models to better account for heterogeneity (Nakagawa et al., 2022). Subsequently, Egger’s regression test was extended to account for our three-level models by including the SE as a moderator in our models.

However, multiple simulation studies have shown poor performance of SEs as moderators (Deeks et al., 2005; Fernández-Castilla et al., 2021; Macaskill et al., 2001; Peters et al., 2006), including when using ORs as effect sizes (Peters et al., 2006). Research attributes the poor performance to issues of correlation between effect size and SEs, subsequently increasing Type Ι errors and artefactual publication bias (Nakagawa et al., 2022). In contrast, some of the aforementioned simulation studies (Fernández-Castilla et al., 2021; Peters et al., 2006) have found inverse sample size as a moderator can outperform SE by mitigating the risk of Type Ι errors due to the independence of effect sizes and inverse sample sizes. Subsequently, we chose to include both moderators for transparency and comparison.

We initially proposed using the trim-and-fill method (Duval & Tweedie, 2000) to identify and correct publication bias. However, we did not anticipate the level of heterogeneity, nesting, and nonindependence of effect sizes in our analyses. The trim-and-fill method has not yet been generalized to models with more than two levels and it was thus not possible to complete this analysis in our multilevel meta-analyses whilst accounting for nonindependence of effect sizes. We have chosen to share the results of the summary effects after the trim-and-fill analysis for all five models (Table 7), but we recommend notable caution when seeking meaningful interpretation of the three-level models where nonindependence of effect sizes has been ignored in line with extant recommendations (Peters et al., 2007; Rodgers & Pustejovsky, 2021; Terrin et al., 2003).

## Results

There was a core focus on violence against children (VAC) ACEs, with studies focusing less on ACEs relating to in-care populations and household/parental-related ACEs (e.g., parental imprisonment, parental substance (mis)use, parental absence including death).

All six key mental health outcomes were represented in the final 62 studies with many studies including multiple key outcomes: depression (*N* = 48 studies), anxiety (*N* = 21), suicidality (*N* = 12), PTSD (*N* = 7), psychotic-like experiences (*N* = 7), and self-harm (*N* = 1). It should be noted that self-harm was measured as an outcome variable in many large-scale longitudinal cohort studies but, unfortunately, was only considered as a lifetime measurement in all studies bar one. For example, in a study using data from the Avon Longitudinal Study of Parents and Children (ALSPAC), Dantchev et al. (2019) included the item “Have you ever hurt yourself on purpose in any way (e.g., by taking an overdose of pills, or by cutting yourself)?” to measure self-harm at 24 years. While lifetime measures provide an understanding of how mental health conditions develop throughout the life course, there is no guarantee that the outcome was present in adulthood as the condition may have both started and ended during childhood or adolescence. We also observed that psychosis was represented less than expected, again due to issues with the outcome not being explicitly measured in adulthood. For example, while some studies were included in this review that used The ALSPAC cohort to explore outcomes other than psychosis (Stapinski et al., 2014), there were other papers analyzing ALSPAC cohort data where psychotic experiences at 18 years old were included as an outcome, but the questions captured any psychotic experiences occurring between 12 and 18 years old (e.g., Wolke et al., 2014) and thus were excluded. The same could be said for PTSD, either with lifetime prevalence being used in adult measures of PTSD (e.g., Nikulina et al., 2011) or because it was grouped as an anxiety disorder alongside generalized anxiety disorder, panic disorder and obsessive-compulsive disorder without a disaggregated effect size presented (e.g., Copeland et al., 2013).

### Study Quality

The overall quality of studies, assessed by the star-based NOS for Cohort Studies (Wells et al., 2000), was high. This is attributed to most studies being large-scale, prospective cohort studies. However, as stated in our protocol for the systematic review (Thurston et al., 2023), total star ratings for each study will not be reported due to limitations associated with total quality scores (Higgins et al., 2023). Instead, we will provide a detailed summary of where studies were (un)successful in the quality assessment. Table 1 refers to the eight categories of quality measured in the NOS and how many studies received a star in each category.

**Table 1.** Summary of the NOS Quality Assessment by Assessment Category.

Quality Assessment Category	No. Studies with Star for Each Item (%)
Representativeness of exposed cohort	62 (100)
Selection of the nonexposed cohort	62 (100)
Was the follow-up long enough for outcomes to occur	62 (100)
Comparability of cohorts based on design or analysis	
(1^st^ star)	59 (95)
(2^nd^ star)	51(82)
Adequacy of follow-up cohorts	58 (94)
Ascertainment of exposure	32 (52)
Demonstration that outcome of interest was not present at start of study	15 (24)
Assessment of outcome	6 (10)

One of the most common limitations with the papers in this review was a lack of “Demonstration that the outcome of interest was not present at start of study.” Research suggests that after controlling for the same mental health outcome in childhood and/or adolescence, relationships between ACEs and adult mental health outcomes may no longer be significant (Ackard et al., 2007). Because psychiatric symptoms in childhood often manifest differently or fall outside adult diagnostic categories, broader adjustments—such as for internalizing/externalizing problems or any Axis I disorder—may offer a more valid approach to accounting for preexisting mental health difficulties (Costello & Angold, 2016). For this reason, studies that included these broader adjustments were also awarded credit in our quality appraisal, recognizing their efforts to account for confounding while also reflecting developmental nuance.

While NOS (Wells et al., 2000) guidelines do not assign stars to any self-reporting of exposure or outcome, we want to highlight that in the context of VAC and other childhood adversity exposures, child and adolescent self-reporting is widely regarded as a reliable method for obtaining data (Meinck et al., 2023; Oláh et al., 2023). Future reviews may reconsider whether NOS is an appropriate quality scale to measure study quality in the fields of ACEs or VAC.

In general, the studies included were vastly heterogeneous in nature. The heterogeneity could be seen in various study aspects such as the way the studies define/conceptualize each mental health outcome (e.g., psychotic symptomatology (Boden et al., 2016) vs. 12-month nonaffective psychosis [Abajobir et al., 2017]); the ages at which ACEs and mental health data are collected; the study designs (e.g., longitudinal birth cohort studies vs. longitudinal data linkage or case-control studies); the main sources of reporting for ACEs and mental health outcomes (e.g., self-reported, informant-reported, medical examiner observation, official records); and the way the studies define and measure the ACEs. For example, studies may use in their analyses single ACE effects such as sexual abuse or neglect (Kisely et al., 2018) versus combined ACE effects such as “two+ episodes of substantiated maltreatment” (Abajobir et al., 2017), or measures that emphasize age of ACE onset such as “bullying at 10 years old” (Lereya et al., 2015) versus measures that emphasize severity of the adversity such as “occasional” or “frequent” peer victimization (Stapinski et al., 2014). Given the study heterogeneity discussed, the results will be separated by mental health outcome.

### Meta-Analysis Results

We used unadjusted effect sizes as opposed to adjusted effect sizes in our meta-analyses. This was because studies adjusted for notably different combinations of covariates and thus the adjusted ORs were not meaningfully comparable. Despite the heterogeneity in which variables were controlled, we felt it was important to explore whether there was a moderating effect of controlling for the relevant adult mental health outcome in childhood.

#### 2 vs 3-Level Meta-Analyses

Fit indices were used to explore whether two- or three-level models were most appropriate for each of the five meta-analyses. We found the three-level model provided a significantly better fit for depression, anxiety, and suicidality mental health outcomes and fit indices pointed toward the use of a two-level model for the PTSD and Psychosis models. Model fit indices are available at https://osf.io/4cxwe.

#### Overall Effect Size

Table 2 displays the pooled effects for each meta-analytic model. The results suggest that adults who were exposed to ACEs have significantly higher odds of developing depression, anxiety, PTSD, psychosis, and suicidality compared to adults who have not been exposed to adversity in childhood. Contour enhanced funnel plots with unadjusted ORs and their 95% prediction intervals for the associations between ACEs and adult mental health outcomes are available at https://osf.io/ma5ny.

**Table 2.** Summary Effect for All Models.

Mental Health Outcome	Number of Studies	Number of Unadjusted Effect Sizes	Mean OR (SE) before trim-and-fill	95% CI	95% PI
Depression	28	97	1.61 (.08) ***	1.45; 1.76	0.84; 2.37
Anxiety	14	61	1.78 (.17) ***	1.45; 2.11	0.61; 2.96
Suicidality	7	22	1.28 (.07) ***	1.13; 1.43	0.95; 1.61
PTSD	4	14	2.26 (.24) ***	1.75; 2.77	1.15; 3.36
Psychosis	5	17	1.34 (.10) ***	1.13; 1.54	0.09; 1.78

*Note.* OR = Odds Ratio; SE = Standard Error; CI = Confidence Interval; PI = Prediction Interval.

*** *p* < .0001.

#### Variance of the Overall Effect Size: Distribution of Variance

Table 3 provides the results displaying the total heterogeneity (variance) for each model and for the three-level models, also displays the distribution of variance across levels. The I² plots showing the distribution of variance across the three-level models for depression, anxiety, and suicidality can be found at https://osf.io/ern29, and profile plots for all five models can be found at https://osf.io/5js4k.

**Table 3.** Total Variance for All Models.

Mental Health Outcome	Within-cluster Heterogeneity (%)	Between-cluster Heterogeneity (%)	Total Heterogeneity I2 (%)
Depression	28.38	61.49	89.87
Anxiety	16.17	72.4	88.57
Suicidality	6.26	50.57	56.83
PTSD	—	—	39.53
Psychosis	—	—	25.36

*Note*. For two-level models, only a percentage of total heterogeneity is provided in the model output.

#### Publication Bias

We used funnel plots (found at https://osf.io/ma5ny) to graphically assess and identify publication bias, but caution is advised when interpreting them for models with <10 studies (PTSD, psychosis, and suicidality). Forest plots for all five models can be found at https://osf.io/ypvg7.

Table 4 displays the results from the classic Egger’s test for two-level PTSD and psychosis models, showing significant risk of publication bias in both models. This agreed with the funnel plots for PTSD and psychosis available at https://osf.io/ma5ny, which both display asymmetry by a suggestion of missing studies on the left-hand side of the plots and a disproportionate representation of studies that had large, positive effects with smaller SEs and had an absence of smaller, nonsignificant, and/or negative effects. The extended Egger’s regression test results for Anxiety, Depression and Suicidality models (Table 5) confirmed the estimates of the SE were significant in all three models. Please note that in this extension, the slope (β1), not the intercept, is the bias indicator (Egger et al., 1997). All models exhibited a positive slope and suggested the potential overestimation of true effects. However, when using inverse sample size as a moderator (Table 6), the results conflicted with those using SE, highlighting Egger’s extended regression test may vary as a function of extraneous factors (e.g., choice of moderator, the number of studies included, high between-study heterogeneity, and the magnitude of effect sizes [including small study effect sizes]) (Fernández-Castilla et al., 2021; Peters et al., 2006; Sterne et al., 2000).

**Table 4.** Results From the Classic Egger Regression Test for the Two-Level Meta-Analytic Models.

Mental Health Outcome	Estimate (b)	*t* (*df*)	Lower CI (95%)	Upper CI (95%)	*p*
PTSD	1.23	8.53 (12)	1	1.46	<.0001
Psychosis	1.05	2.79 (15)	0.82	1.28	.0137

**Table 5.** Results from the Three-Step Extension of Egger’s Regression Test with Standard Error as the Moderator.

Mental Health Outcome	Egger’s Estimate (β_0_)	*t*(*df*) (β_0_)	Lower CI (β_0_)	Upper CI (β_0_)	Egger’s Estimate (β_1_)	*t* (*df*) (β_1_)	Lower CI (β_1_)	Upper CI (β_1_)
Depression	1.22	14.59 (95) ***	1.05	1.39	1.29	6.32 (95) ***	0.88	1.69
Anxiety	1.3	9.17 (59) ***	1.01	1.58	1.34	5.07 (59) ***	0.81	1.87
Suicidality	1.04	33.05 (20) ***	0.98	1.11	1.44	5.03 (20) ***	0.84	2.03

*Note*. Estimate = estimate of the standard error.

*** *p* < .0001.

**Table 6.** Results From the Three-Step Extension of Egger’s Regression Test with Inverse Sample Size as the Moderator.

Mental Health Outcome	Egger’s Estimate (β_0_)	*t* (*df*) (β_0_)	Lower CI (β_0_)	Upper CI (β_0_)	Egger’s Estimate (β_1_)	*t* (*df*) (β_1_)	Lower CI (β_1_)	Upper CI (β_1_)
Depression	1.7	15.95 (95) ***	1.49	1.92	−91.20	−1.41 (95)	−219.26	36.86
Anxiety	1.76	5.75 (59) ***	1.15	2.37	41.96	0.12 (59)	−685.95	769.83
Suicidality	1.23	9.39 (20) ***	0.96	1.51	265.55	0.50 (20)	−844.09	1375.19

*Note*: Estimate = estimate of the inverse sample size.

*** *p* < .0001.

Trim-and-fill analysis results (Table 7) are presented for all five models, but as mentioned prior, we recommend caution when interpreting three-level models that ignore nonindependence of effect sizes.

**Table 7.** Summary Effect for All Models after Trim-and-Fill Analysis.

Mental Health Outcome	Mean Unadjusted OR (SE) after trim-and-fill	95% CI	95% PI	Total Heterogeneity I2 (%)
Depression	1.46 (0.06) ***	1.34; 1.58	0.49; 2.43	92.07
Anxiety	1.46 (0.10) ***	1.26; 1.66	0.12; 2.80	89.94
Suicidality	1.17 (0.07) ***	1.03; 1.32	0.66; 1.69	75.05
PTSD	1.87 (0.18) ***	1.52; 2.22	1.16; 2.58	18.56
Psychosis	1.28 (0.10) ***	1.09; 1.47	0.87; 1.69	20.42

Note. OR = Odds Ratio; SE = Standard Error; CI = Confidence Interval; PI = Prediction Interval.

*** *p* < .0001.

### Moderator Analysis

Moderator analyses examined the separate moderating effects of year of publication, individual ACE type (e.g., parental separation, household conflict), broader/umbrella ACE type (e.g., household dysfunction), ACE count (single vs multiple), location, ACE source of reporting, and study design on the relationship between ACEs and adult depression, anxiety, PTSD, psychosis, and suicidality. Aligning with the way a vast body of literature has conceptualized and categorized ACEs, we also ran models exploring the moderating effects of ACE types grouped into the main groups: Child Abuse and Neglect vs other ACEs, Household Dysfunction vs other ACEs (Cavanaugh, 2016; Felitti et al., 1998), and aligning with more recent literature that has acknowledged bullying victimization as an ACE (Arseneault, 2018; Vaswani, 2019), bully victim versus other ACEs.

When exploring the effect of individual ACE types, we selected “Parental Separation” as the reference category and “Household Dysfunction” as the reference when looking at broader categories of ACE types. These were chosen as the reference categories as research suggests ACEs in the household dysfunction category may have less severe impacts on mental ill-health compared to ACEs where there is a maltreatment component such as bullying or child abuse (Negriff, 2020). Furthermore, studies have suggested that the long-term mental health consequences associated with household-related childhood adversity can be fully mediated by the presence of childhood maltreatment (Clemens et al., 2019). Please note that for some univariate moderator analyses, there were insufficient data or too few studies to allow for analysis. For a detailed description of how each moderator was grouped, please see Supplemental Appendix H. Table showing the results of overall univariate model effects for moderator analyses per mental health outcome can be found in Supplemental Appendix I.

#### Depression

Tables showing full moderator analysis results can be found in Supplemental Appendix J. There was an overall moderating effect of ACE type when looking at individual ACE effects: *F* (15, 81) = 4.13, *p* < .0001. Two of the three experiences that involved bullying victimization were significantly different from the reference category parental separation, except for bullying victimization measured on its own, which was nonsignificant (*p* = .0526).

Furthermore, the categories “child abuse and neglect,” household/parental conflict and cumulative ACEs also significantly differed from the reference category. The results suggests that childhood bullying victimization (while also perpetrating bullying): β_1_ = .92 (95% CI [0.09, 1.76]), *t*(81) = 2.19, *p* = .0311; being both a bully victim and experiencing childhood maltreatment: β_1_ = 1.66 (.42; 2.89), *t*(81) = 2.67, *p* = .0092; experiencing child abuse and neglect: *β*_1_ = .44 (.15; .72), *t*(81) = 3.06, *p* = .003; experiencing household conflict in childhood: *β*_1_ = .64 (.20; 1.08), *t*(81) = 2.91, *p* = .0046; and experiencing cumulative ACEs: β_1_ = .51 (.1; .92), *t*(81) = 2.45, *p* = .0163, all result in significantly higher odds of presenting with adult depressive symptoms than the mean effect of parental separation on depressive symptoms: β_0_ = 1.31 (1.06; 1.56), *t*(81) = 10.40, *p* < .0001. When comparing the moderating effect of all other ACEs to Household Dysfunction, experiencing household dysfunction resulted in significantly lower odds of presenting with depressive symptoms in adulthood: β_1_ = −.23 (95% CI [−0.43, −0.03]), *t*(95) = −2.24, *p* = .0227, than the mean effect of experiencing any other ACE types on depressive symptoms: *β*_0_ = 1.68 (1.5; 1.85), *t*(95) = 18.73, *p* < .0001. No other moderators had a significant overall model effect, but when examining location, results showed that participants in the study conducted in South America, *β*_1_ = .80 (.05; 1.56), *t*(92) = 2.12, *p* = .0368, had significantly higher odds of presenting with adult depressive symptoms than the mean effect of the reference category, North America *β*_1_ = 1.60 (1.39; 1.82), *t*(92) = 14.78, *p* < .0001.

#### Anxiety

Tables showing full moderator analysis results can be found in Supplemental Appendix K. There was a moderating effect of location as the results of the omnibus test point toward a significant moderating effect: *F* (3, 57) = 3.75, *p* = .0158. Grouping studies by continent showed that participants in the study conducted in Africa, *β*_1_ = −86 (−1.58; −.13), *t*(57) = −2.35 *p* = .022, had significantly lower odds of presenting with adult anxiety symptoms than the mean effect of the reference category, North America *β*_0_ = 1.81(1.46; 2.16), *t*(57) = 10.28, *p* < .0001.However, we stress this should be only viewed as a preliminary finding given the limited sample size. No other moderators were significant.

#### Suicide

Tables showing full moderator analysis results can be found in Supplemental Appendix L. When comparing the moderating effect of all other ACEs to Child Abuse and Neglect, the results of the omnibus test point toward a significant moderating effect: *F* (1, 20) = 10.61, *p* = .0039. The model results showed experiencing child abuse and neglect resulted in significantly lower odds of presenting with suicidality in adulthood, *β*_1_ = −.29 (95% CI [−0.47, −0.1]), *t*(20) = −3.26, *p* = .0039, than the mean effect of experiencing any other ACE types on suicidality, *β*_0_ = 1.42(1.25; 1.59), *t*(20) = 17.43, *p* < .0001. No other moderators were significant.

#### PTSD

Tables showing full moderator analysis results can be found in Supplemental Appendix M. There was a moderating effect of location as the results of the omnibus test point toward a significant moderating effect: *F* (1, 12) = 13.25, *p* = .0034. Compared to participants in studies conducted in North America β_0_ = 1.74 (95% CI [1.44, 2.03]), *t*(12) = 12.90, *p* < .0001, participants in studies conducted in Australia/Oceania exhibited significantly higher odds of presenting with PTSD symptoms in adulthood *β*_1_ = 1.22 (.49; 1.94), *t*(12) = 3.64, *p* = .0034. No other moderators were significant.

#### Psychosis

Tables showing full moderator analysis results can be found in Supplemental Appendix N. No moderators were significant.

## Discussion

Table 8 provides a summary of critical findings. This study aimed to quantitatively summarize longitudinal research examining the relationship between prospectively measured ACEs and six adult mental illnesses that have been commonly and strongly associated with ACEs in literature: depression, anxiety, PTSD, suicidality, self-harm, and psychotic-like experiences. To fulfil this aim, we conducted separate meta-analyses where possible for these outcomes; two of which were two-level meta-analyses (for psychosis and PTSD) and three of which were three-level meta-analyses (for depression, anxiety, and suicide). Adult self-harm was only represented in one study, preventing meaningful narrative synthesis and meta-analysis. These findings add to a well-established evidence base but importantly extend it by focusing exclusively on prospective measurement of ACEs during childhood and mental health outcomes in adulthood (excluding lifetime measures), using a diverse variety and broad number of databases, included numerous expanded ACEs in the conceptualization and definition of ACEs, widened the mental health scope beyond diagnoses, and included gray literature. This design choice strengthens the temporal inference of associations and avoids the known biases of retrospective ACE reporting, while also allowing a more rigorous examination of moderators that may influence these relationships.

**Table 8.** Table Showing a Summary of Critical Findings.

Summary of Critical Findings
1. Sixty-two studies were included in the final review; 39 were meta-analyzed. 2. Most studies were from the USA with a significant uneven global distribution (95% of studies being from high-income countries). 3. The quality of studies was overall high, but studies were vastly heterogenous in nature making meaningful comparisons across studies difficult. 4. ACEs were associated with an increased likelihood of depression, anxiety, suicidality, PTSD and psychosis. 5. ACE type and location moderated the effects found for depression, anxiety and suicidality but there were no significant moderators found for PTSD or psychosis. 6. This review underscores ACEs as critical risk markers for adult mental ill-health. 7. We call for better standardization in ACE conceptualization, measurement tools and analytic frameworks but stress specific structural, economic, societal, and cultural contexts should remain respected and considered. 8. This review also highlights the urgent need for funding to allow longitudinal ACE and adult mental health research to be conducted in diverse global contexts, particularly low- and middle-income contexts.

For all mental health outcomes examined, pooled unadjusted ORs indicated significantly increased odds among those who experienced ACEs compared to those who did not. Associations were strongest between ACEs and adult PTSD, OR 2.26 (95% CI [1.75, 2.77]). However, we caution against overinterpreting this finding given the limited number of studies included in the PTSD model (ki = 4, *N* ES = 14). Most robust evidence was available for depression and anxiety, both of which demonstrated consistent patterns of increased odds among individuals who experienced ACEs. While psychosis and suicidality also showed significant associations with ACEs, these models included fewer studies which limited the precision and generalizability of pooled estimates. All five models demonstrated significant heterogeneity, which is unsurprising given the diverse designs, populations, and analytical choices across studies.

We propose the risk of missing high-quality research not published in English is very low given existing evidence that most work exploring the prevalence and impact of ACEs has been conducted in HICs, particularly in Europe and North America (Soares et al., 2016). In addition, most longitudinal cohort studies have been disproportionately funded and conducted in HICs (Victora & Barros, 2012) where English is either the first language or the language in which research output is published.

Although our methods aimed to support greater inclusion of studies from LMICs through the inclusion of gray literature and regionally indexed databases, only a small number of eligible LMIC studies met our final criteria. When conducting our title and abstract screening, many studies from LMICs were screened but did not meet our stringent inclusion criteria. For example, they were predominantly cross-sectional in design and measured ACEs retrospectively (e.g., LeMasters et al., 2021; Masiano et al., 2022; Ramiro et al., 2010) or they were not focused on associations with our chosen outcomes (e.g., Bhengu et al., 2020). Some longitudinal cohorts in LMICs are still in their earlier years, and adult data are not yet available for their participants, such as the nationally representative Longitudinal Cohort Study on the Filipino Child (USC-Office of Population Studies Foundation, 2019) aiming to follow 5000 children who were aged 10 in 2016 for 15 years, or the INTERRUPT_VIOLENCE longitudinal study in South Africa (Meinck et al., 2023) that is building on an existing two-wave cohort study where ACEs were measured in childhood. The lack of longitudinal cohort data in LMICs highlights the urgent need for more research into the prevalence and long-term mental health impacts of ACEs in these contexts. Without this, current global evidence remains geographically skewed and may underrepresent culturally specific adversities or contextually relevant risk pathways.

Our moderator analyses explored whether study-level factors moderated the associations between ACEs and adult mental health outcomes. Specifically, we examined the separate moderating effects of year of publication, individual ACE type (e.g., parental separation, household conflict), broader ACE type (e.g., household dysfunction vs. other ACEs), ACE count (single vs. multiple), location, source of ACE reporting, and study design. These variables were chosen to reflect how ACEs are commonly grouped and conceptualized in the literature (Cavanaugh, 2016; Felitti et al., 1998), as well as to incorporate more contemporary understandings of adversity such as bullying victimization (bully vs. other ACEs) (Arseneault, 2018; Vaswani, 2019). While some moderators showed significant effects in models for depression and PTSD, the moderator analyses for anxiety, suicidality, and psychosis yielded fewer or nonsignificant findings. This may reflect limited power due to the smaller number of studies per outcome or substantial between-study heterogeneity. In addition, due to the small number of LMIC studies (*n* = 3), we were unable to include income classification as a moderator. Although we were unable to statistically examine other sources of heterogeneity—such as variation in ACE measurement tools, age of ACE assessment, or whether outcomes were symptom-based or diagnostic—these differences likely contributed to variability in effect sizes. The timing and method of outcome measurement in adulthood may also have influenced findings, particularly where studies used broad or undifferentiated mental health categories. These inconsistencies highlight the challenge of establishing precise associations between specific ACEs and mental health outcomes across diverse study contexts and measurement designs.

### Implications of Findings

A summary of implications for practice, policy, and research is shown in Table 9. While there is growing interest in ACE screening in primary care, inpatient psychiatric units, and mental health clinics due to the well-established relationship between ACEs and poor mental health, we do not recommend screening for ACEs as a predictive tool for mental health diagnoses in adulthood. ACEs screening has been found to have some clinical utility in predicting risk of poor mental health in adolescents, it may not be as accurate or fair in predicting poor mental health outcomes across different sociodemographic factors, including gender and ethnicity (Cohen & Choi, 2022). In addition, cumulative ACE scores have been found to have poor predictive accuracy for later life mental ill-health (Baldwin et al., 2021). However, clinicians may still find it beneficial to consider ACE exposure as part of a broader biopsychosocial assessment to support in deciding the best individualized mental health prevention and treatment options.

**Table 9.** Table Summary of the Implications for Practice, Policy, and Research.

Summary of Implications for Practice, Policy and Research
1. Pooled odds ratios indicated those who experienced ACEs had significantly higher odds of presenting with mental ill-health than those who had not experienced ACEs. This result was found across all five mental health outcomes that were meta-analyzed: depression, anxiety, PTSD, psychotic-like experiences and suicidality. 2. When deciding the best mental health treatment for adults in various healthcare contexts, screening for ACEs may support professionals and patients understand the possible etiology of their mental health condition/s. 3. Our findings support the continued development of prevention interventions for violence against children when attempting to reduce mental illness’ contribution to the global burden of disease. 4. There was vast heterogeneity across studies which meant drawing meaningful comparisons was difficult. Future research should endeavor to create better standardization of ACE conceptualization, measurement tools, and analytic frameworks while respecting specific structural, economic, societal, and cultural contexts. 5. We urgently recommend more funding toward prospective longitudinal research in LMICs with a core focus on exploring the social determinants of mental health outcomes, including ACEs.

Despite the consensus that ACEs co-occur to impact long-term outcomes (M. Allen & Donkin, 2015), a recent evidence review of interventions to prevent ACEs (Di Lemma et al., 2019) found most existing interventions target or respond to individual ACEs such as child abuse. Our findings suggest specific ACEs involving maltreatment and/or violence components—such as physical abuse and bullying victimization—seem to have stronger associations with adult depression than other childhood adversities. Subsequently, our findings support the continued development of prevention programs for VAC in reducing mental illness’ contribution to the global burden of disease. This aligns with well-established findings that parenting interventions are key factors for reducing and preventing specific ACEs such as child abuse (Asmussen et al., 2020; Chen & Chan, 2016; Di Lemma et al., 2019), which may in turn reduce the risk of later life mental illness. Our recommendation of continued focus on violence prevention extends to anti-bullying initiatives. For example, Fraguas et al. (2021) conducted a meta-analysis of 69 randomized clinical trials assessing school anti-bullying interventions. Findings concluded anti-bullying interventions had a substantial population impact of reduced bullying and improved mental health problems. More broadly, policies aimed at preventing VAC and improving social welfare systems have the potential to act as “developmental accelerators,” a term coined in 2017 by the United Nations Development Programme. Developmental accelerators concurrently work toward multiple sustainable development goals at once by addressing and reducing ACEs and improving long-term well-being and relationships. Importantly, these policies must be locally adapted and contextually appropriate, particularly in LMICs, to ensure they reflect the types of adversities children are most likely to face in those settings.

While our findings support the relevance of ACEs as risk markers across a range of mental health outcomes, the strength and consistency of associations varied. Although the direction of associations was consistently positive across all outcomes, the magnitude of associations varied, with PTSD showing the largest pooled effect size, followed by depression and anxiety, and smaller yet significant estimates for suicidality and psychotic-like experiences. Depression and anxiety were the most frequently studied outcomes and showed robust, consistent associations with ACEs. In contrast, PTSD, suicidality, and psychotic-like experiences were examined in fewer studies and displayed wider variability in effect sizes. The evidence base on self-harm was especially limited, with only one study meeting inclusion criteria, despite self-harm being a common and high-burden issue in the general population (McManus et al., 2019). Similarly, psychotic-like experiences are increasingly recognized as clinically significant but remain underrepresented in longitudinal ACE research (van Os et al., 2009). These gaps point to a need for future longitudinal research to give greater attention to a broader spectrum of mental health outcomes, including those that are less commonly studied but highly prevalent and burdensome. Methodologically, future studies should also strive to improve the precision of outcome measurement—for example, by clearly distinguishing between symptomatology and clinical diagnoses, using validated tools, and reporting on the timing and chronicity of mental health outcomes in adulthood. Without such improvements, the field will remain limited in its ability to draw meaningful comparisons and inform prevention or treatment strategies tailored to specific mental health conditions.

We have been unable to conclude what ACEs may be most strongly associated with the other mental health outcomes anxiety, PTSD, suicide, and psychosis. We could infer that our lack of findings may be due to limitations in the evidence base that did not allow for definitive testing, such as too few studies being included in the moderator analysis, a lack of statistical power, and/or high levels of heterogeneity. While we found no observable significant differences in the associations between individual ACEs and these mental health outcomes, prior literature has shown that the type of ACEs children are exposed to differentially impact long-term health outcomes (Westermair et al., 2018). To allow future meta-analyses to conduct definitive testing of what ACEs may be most strongly associated with the aforementioned mental health outcomes, studies first need to focus on addressing the large heterogeneity and methodological inconsistencies across studies, including variation in ACE conceptualization, measurement, and the disparity in the ways ACEs are grouped when analyzed. Greater use of harmonized measures and clearer reporting of effect size adjustments will also be a key for improving future meta-analytic syntheses.

### Limitations

Despite ACEs being measured in childhood, which should have allowed for better assessment of developmental changes and the timing and chronicity of ACEs (Arjas & Parner, 2004; Salo et al., 2021), heterogeneity and missing data prevented analysis of ACE onset age and chronicity. Both factors are known to influence health outcomes (English et al., 2005; Masten & Cicchetti, 2010; Schalinski et al., 2016). Furthermore, genetic variations impacting lifetime psychopathology risk and ACEs–mental health associations were unaccounted for. In addition, prospective longitudinal studies provide an opportunity for temporal patterning of events and confounding variables to be adjusted for at each time point (Connolly, 2018). However, most studies (76%) did not control for the same mental health outcome in childhood, limiting causal inferences. This omission makes it difficult to distinguish whether observed effects represent new onset adult psychopathology or the continuation of early-life conditions.

Our inclusion criteria focused on general community samples, excluding special subpopulations where ACE–mental health associations may be qualitatively different. This decision aimed to facilitate more meaningful cross-study comparisons, but may have inadvertently excluded populations with the strongest associations, such as inpatients in psychiatric hospitals or people currently in prison.

Geographic variability in ACE prevalence and associations to health outcomes is well-documented (M. Allen & Donkin, 2015; Bellis et al., 2023). However, our moderator analysis of location was constrained by the fact that 95% of included studies were conducted in HICs, reducing variability for comparison.

Our inclusion criteria stipulated that ACEs had to have been measured during childhood (<18 years), and while this had aforementioned benefits, in doing so we excluded studies that were prospective in nature but that collected retrospective ACE data in adulthood that is, research of similar design to the original ACE study (Felitti et al., 1998) such as studies using the UK biobank data to explore associations between childhood adversity and adult mental health outcomes (S. F. Allen et al., 2023).

Disparities in ACE conceptualization, ACE and mental health measurement, and statistical approaches contributed to heterogeneity across studies. Studies used varied ACE reporting methods, including self-reports, informant reports, and official records such as court documents, each introducing distinct biases (Auersperg et al., 2019; Chan, 2008; Cunningham et al., 2016; Hampton & Newberger, 1985; Hunt et al., 2003; Kisely et al., 2018; Pinto et al., 2014). As a result of this inconsistency, we were unable to examine the effect of the number of ACEs as a moderator in our meta-analysis. Most studies did not report comparable or harmonized ACE counts. Instead, they presented study-specific thresholds, binary groups, or disaggregated exposures. The heterogeneity in analytic techniques and reporting methods necessitated the conversion of several effect size estimates to ORs. However, we acknowledge that these conversions may have introduced bias or imprecision due to approximations involved. The heterogeneity between study estimates is further evidenced by the vast dispersion in prediction estimates for each of the five models (see Table 7). Finally, while our use of a three-level meta-analytic model and moderator analyses allowed us to account for between- and within-study variability, we did not conduct formal sensitivity analyses (e.g., leave-one-out tests). Future reviews should consider incorporating such robustness checks to better assess the stability of pooled estimates.

The methodological limitations highlight the need for improved standardization of ACE conceptualization, measurement, and analytic frameworks. The authors acknowledge that any standardization should remain context-specific, and stress that specific structural, economic, societal, and cultural contexts should remain respected and considered. For example, ACE measurement could benefit from better standardization of a set of core indicators while also ensuring contextually diverse and context-specific ACEs are captured in Supplemental Appendices.

## Conclusion

This systematic review and meta-analysis reaffirms the importance of identifying ACEs as risk markers for later poor adult mental health, including depression, anxiety, PTSD, psychotic-like experiences, and suicidality. While confirming consistency in the strong association between ACEs and adult mental ill-health across outcomes, our findings also highlight significant methodological and analytic inconsistencies between studies. Our findings provide a compelling case for the field to make meaningful progress toward greater alignment in how ACEs are conceptualized, measured, and analyzed. Future studies must also expand beyond HICs to capture the full global picture of ACEs and their long-term consequences. Until these challenges are addressed, the field may continue to face limitations in drawing robust inferences and making equitable, evidence-based policy recommendations.

## Supplemental Material

sj-docx-1-tva-10.1177_15248380251358223 – Supplemental material for Prospective Longitudinal Associations Between Adverse Childhood Experiences and Adult Mental Health Outcomes: Systematic Review and Meta-Analysis Supplemental material, sj-docx-1-tva-10.1177_15248380251358223 for Prospective Longitudinal Associations Between Adverse Childhood Experiences and Adult Mental Health Outcomes: Systematic Review and Meta-Analysis by Christina Thurston, Aja Louise Murray, Hannabeth Franchino-Olsen, Mpho Silima, Chad Lance Hemady and Franziska Meinck in Trauma, Violence, & Abuse

## References

[bibr1-15248380251358223] Abajobir A. A. Kisely S. Scott J. G. Williams G. Clavarino A. Strathearn L. Najman J. M. (2017). Childhood maltreatment and young adulthood hallucinations, delusional experiences, and psychosis: A longitudinal study. Schizophrenia Bulletin, 43(5), 1045–1055. 10.1093/schbul/sbw175 28338760 PMC5581886

[bibr2-15248380251358223] Ackard D. M. Eisenberg M. E. Neumark-Sztainer D. (2007). Long-term impact of adolescent dating violence on the behavioral and psychological health of male and female youth. The Journal of Pediatrics, 151(5), 476–481. 10.1016/j.jpeds.2007.04.034 17961688

[bibr3-15248380251358223] Allen M. Donkin A. (2015). The impact of adverse experiences in the home on children and young people. Institute of Health Equity. https://www.instituteofhealthequity.org/resources-reports/the-impact-of-adverse-experiences-in-the-home-on-children-and-young-people

[bibr4-15248380251358223] Allen S. F. Gilbody S. Atkin K. van der Feltz-Cornelis C. M. (2023). The associations among childhood trauma, loneliness, mental health symptoms, and indicators of social exclusion in adulthood: A UK Biobank study. Brain and Behavior, 13(4), e2959. 10.1002/brb3.2959 PMC10097065 36922902

[bibr5-15248380251358223] Arjas E. Parner J. (2004). Causal reasoning from longitudinal data. Scandinavian Journal of Statistics, 31(2), 171–187. 10.1111/j.1467-9469.2004.02-134.x

[bibr6-15248380251358223] Arseneault L. (2018). Annual research review: The persistent and pervasive impact of being bullied in childhood and adolescence: Implications for policy and practice. Journal of Child Psychology and Psychiatry, 59(4), 405–421. 10.1111/jcpp.12841 29134659 PMC6542665

[bibr7-15248380251358223] Asmussen K. Fischer F. Drayton E. McBride T. (2020). Adverse childhood experiences: What we know, what we don’t know, and what should happen next. Early Intervention Foundation. https://www.eif.org.uk/report/adverse-childhood-experiences-what-we-know-what-we-dont-know-and-what-should-happen-next

[bibr8-15248380251358223] Assink M. Wibbelink C. (2016). Fitting three-level meta-analytic models in R: A step-by-step tutorial. The Quantitative Methods for Psychology, 12, 154–174. 10.20982/tqmp.12.3.p154

[bibr9-15248380251358223] Auersperg F. Vlasak T. Ponocny I. Barth A. (2019). Long-term effects of parental divorce on mental health—A meta-analysis. Journal of Psychiatric Research, 119, 107–115. 10.1016/j.jpsychires.2019.09.011 31622869

[bibr10-15248380251358223] Baldwin J. R. Reuben A. Newbury J. B. Danese A. (2019). Agreement between prospective and retrospective measures of childhood maltreatment: A systematic review and meta-analysis. JAMA Psychiatry, 76(6), 584. 10.1001/jamapsychiatry.2019.0097. 30892562 PMC6551848

[bibr11-15248380251358223] Baldwin J. R. Caspi A. Meehan A. J. Ambler A. Arseneault L. Fisher H. L. Harrington H. Matthews T. Odgers C. L. Poulton R. Ramrakha S. Moffitt T. E. Danese A. (2021). Population vs individual prediction of poor health from results of adverse childhood experiences screening. JAMA Pediatrics, 175(4), 385–393. 10.1001/jamapediatrics.2020.5602 33492366 PMC7835926

[bibr12-15248380251358223] Bellis M. A. Hughes K. Ford K. Rodriguez G. R. Sethi D. Passmore J. (2019). Life course health consequences and associated annual costs of adverse childhood experiences across Europe and North America: A systematic review and meta-analysis. The Lancet Public Health, 4(10), e517–e528. 10.1016/S2468-2667(19)30145-8 PMC7098477 31492648

[bibr13-15248380251358223] Bellis M. A. Wood S. Hughes K. Quigg Z. Butler N. (2023). Tackling adverse childhood experiences (ACEs): State of the art and options for action. https://www.ljmu.ac.uk/-/media/phi-reports/pdf/2023-01-state-of-the-art-report-eng.pdf

[bibr14-15248380251358223] Bhengu B. S. Tomita A. Mashaphu S. Paruk S. (2020). The role of adverse childhood experiences on perinatal substance use behaviour in KwaZulu-Natal Province, South Africa. AIDS and Behavior, 24(6), 1643–1652. 10.1007/s10461-019-02661-y 31542877

[bibr15-15248380251358223] Björkenstam E. Vinnerljung B. Hjern A. (2017). Impact of childhood adversities on depression in early adulthood: A longitudinal cohort study of 478,141 individuals in Sweden. Journal of Affective Disorders, 223, 95–100. 10.1016/j.jad.2017.07.030 28735168

[bibr16-15248380251358223] Boden J. M. van Stockum S. Horwood L. J. Fergusson D. M. (2016). Bullying victimization in adolescence and psychotic symptomatology in adulthood: Evidence from a 35-year study. Psychological Medicine, 46(6), 1311–1320. 10.1017/S0033291715002962 26804185

[bibr17-15248380251358223] Burke N. J. Hellman J. L. Scott B. G. Weems C. F. Carrion V. G. (2011). The impact of adverse childhood experiences on an urban pediatric population. Child Abuse & Neglect, 35(6), 408–413. 10.1016/j.chiabu.2011.02.006 21652073 PMC3119733

[bibr18-15248380251358223] Cavanaugh B. (2016). Trauma-informed classrooms and schools. Beyond Behavior, 25(2), 41–46. 10.1177/107429561602500206

[bibr19-15248380251358223] Centers for Disease Control and Prevention. (2019). Preventing adverse childhood experiences: Leveraging the best available evidence. https://www.cdc.gov/violenceprevention/pdf/preventingACES.pdf

[bibr20-15248380251358223] Chan D. (2008). So why ask me? Are self-report data really that bad? In Lance C. E. Vandenberg R. J. (Eds.), Statistical and methodological myths and urban legends: Doctrine, verity, and fable in the organizational and social sciences (pp. 309–336). Routledge.

[bibr21-15248380251358223] Chapman D. P. Whitfield C. L. Felitti V. J. Dube S. R. Edwards V. J. Anda R. F. (2004). Adverse childhood experiences and the risk of depressive disorders in adulthood. Journal of Affective Disorders, 82(2), 217–225. 10.1016/j.jad.2003.12.013 15488250

[bibr22-15248380251358223] Chen M. Chan K. L. (2016). Effects of parenting programs on child maltreatment prevention: A meta-analysis. Trauma, Violence, & Abuse, 17(1), 88–104. 10.1177/1524838014566718 25573846

[bibr23-15248380251358223] Clemens V. Berthold O. Witt A. Sachser C. Brähler E. Plener P. L. Strauß B. Fegert J. M. (2019). Child maltreatment is mediating long-term consequences of household dysfunction in a population representative sample. European Psychiatry, 58, 10–18. 10.1016/j.eurpsy.2019.01.018 30743239

[bibr24-15248380251358223] Cohen J. R. Choi J. W. (2022). Is ACEs screening for adolescent mental health accurate and fair? Prevention Science: The Official Journal of the Society for Prevention Research, 23(7), 1216–1229. 10.1007/s11121-022-01391-3 35778650

[bibr25-15248380251358223] Connolly E. (2018). Evaluating the causal effect of adverse childhood experiences on antisocial behavior and violent victimization: A longitudinal sibling-comparison analysis. College of Criminal Justice, Sam Houston State University. 10.13140/RG.2.2.13330.76481

[bibr26-15248380251358223] Copeland W. E. Wolke D. Angold A. Costello E. J. (2013). Adult psychiatric outcomes of bullying and being bullied by peers in childhood and adolescence. JAMA Psychiatry, 70(4), 419. 10.1001/jamapsychiatry.2013.504 23426798 PMC3618584

[bibr27-15248380251358223] Costello E. J. Angold A. (2016). Developmental epidemiology. In Cicchetti D. (Ed.), Developmental psychopathology (Vol. 1, pp. 1–35). John Wiley & Sons, Ltd. 10.1002/9781119125556.devpsy103

[bibr28-15248380251358223] Cunningham T. Hoy K. Shannon C. (2016). Does childhood bullying lead to the development of psychotic symptoms? A meta-analysis and review of prospective studies. Psychosis, 8(1), 48–59. 10.1080/17522439.2015.1053969

[bibr29-15248380251358223] Dantchev S. Hickman M. Heron J. Zammit S. Wolke D. (2019). The independent and cumulative effects of sibling and peer bullying in childhood on depression, anxiety, suicidal ideation, and self-harm in adulthood. Frontiers in Psychiatry, 10, 651. 10.3389/fpsyt.2019.00651 31616323 PMC6768961

[bibr30-15248380251358223] Deeks J. J. Higgins J. P. T. Altman D. G. ; on behalf of the Cochrane Statistical Methods Group. (2019). Analysing data and undertaking meta-analyses. In Higgins J. P. T. Thomas J. Chandler J. Cumpston M. Li T. Page M. J. Welch V. A. (Eds.), Cochrane handbook for systematic reviews of interventions (Version 6.0, pp. 241–284). John Wiley & Sons, Ltd.

[bibr31-15248380251358223] Deeks J. J. Macaskill P. Irwig L. (2005). The performance of tests of publication bias and other sample size effects in systematic reviews of diagnostic test accuracy was assessed. Journal of Clinical Epidemiology, 58(9), 882–893. 10.1016/j.jclinepi.2005.01.016 16085191

[bibr32-15248380251358223] Di Lemma L. Davies A. Ford K. Hughes K. Homolova L. Gray B. Richardson G . (2019). Responding to adverse childhood experiences. Public Health Wales NHS Trust. https://phw.nhs.wales/news/responding-to-adverse-childhood-experiences-an-evidence-review/responding-to-adverse-childhood-experiences/

[bibr33-15248380251358223] Duval S. Tweedie R. (2000). Trim and fill: A simple funnel-plot-based method of testing and adjusting for publication bias in meta-analysis. Biometrics, 56(2), 455–463. 10.1111/j.0006-341X.2000.00455.x 10877304

[bibr34-15248380251358223] Egger M. Smith G. D. Schneider M. Minder C. (1997). Bias in meta-analysis detected by a simple, graphical test. BMJ, 315(7109), 629–634. 10.1136/bmj.315.7109.629 9310563 PMC2127453

[bibr35-15248380251358223] El Mhamdi S. Lemieux A. Bouanene I. Ben Salah A. Nakajima M. Ben Salem K. al’Absi M . (2017). Gender differences in adverse childhood experiences, collective violence, and the risk for addictive behaviors among university students in Tunisia. Preventive Medicine, 99, 99–104. 10.1016/j.ypmed.2017.02.011 28216378

[bibr36-15248380251358223] English D. J. Graham J. C. Litrownik A. J. Everson M. Bangdiwala S. I. (2005). Defining maltreatment chronicity: Are there differences in child outcomes? Child Abuse & Neglect, 29(5), 575–595. 10.1016/j.chiabu.2004.08.009 15970326

[bibr37-15248380251358223] Fagerland M. W. (2015). Chapter 12—Evidence-based medicine and systematic reviews. In Laake P. Benestad H. B. Olsen B. R. (Eds.), Research in medical and biological sciences (2nd ed., pp. 431–461). Academic Press.

[bibr38-15248380251358223] Felitti V. J. Anda R. F. Nordenberg D. Williamson D. F. Spitz A. M. Edwards V. Koss M. P. Marks J. S. (1998). Relationship of childhood abuse and household dysfunction to many of the leading causes of death in adults: The Adverse Childhood Experiences (ACE) Study. American Journal of Preventive Medicine, 14(4), Article 4. 10.1016/S0749-3797(98)00017-8 9635069

[bibr39-15248380251358223] Fernández-Castilla B. Aloe A. M. Declercq L. Jamshidi L. Beretvas S. N. Onghena P. van den Noortgate W. (2021). Estimating outcome-specific effects in meta-analyses of multiple outcomes: A simulation study. Behavior Research Methods, 53(2), 702–717. 10.3758/s13428-020-01459-4 32808180

[bibr40-15248380251358223] Fraguas D. Díaz-Caneja C. M. Ayora M. Durán-Cutilla M. Abregú-Crespo R. Ezquiaga-Bravo I. Martín-Babarro J. Arango C. (2021). Assessment of school anti-bullying interventions: A meta-analysis of randomized clinical trials. JAMA Pediatrics, 175(1), 44–55. 10.1001/jamapediatrics.2020.3541 33136156 PMC7607493

[bibr41-15248380251358223] Hampton R. L. Newberger E. H. (1985). Child abuse incidence and reporting by hospitals: Significance of severity, class, and race. American Journal of Public Health, 75(1), 56–60. 10.2105/AJPH.75.1.56 3966600 PMC1646161

[bibr42-15248380251358223] Higgins J. P. Thomas J. Chandler J. Cumpston M. Li T. Page M. Welch V. (2023). Cochrane handbook for systematic reviews of interventions version 6.4. https://training.cochrane.org/handbook/current 10.1002/14651858.ED000142 PMC10284251 31643080

[bibr43-15248380251358223] Hughes K. Bellis M. A. Hardcastle K. A. Sethi D. Butchart A. Mikton C. Jones L. Dunne M. P. (2017). The effect of multiple adverse childhood experiences on health: A systematic review and meta-analysis. The Lancet. Public Health, 2(8), Article 8. 10.1016/S2468-2667(17)30118-4 29253477

[bibr44-15248380251358223] Hughes K. Ford K. Kadel R. Sharp C. A. Bellis M. A. (2020). Health and financial burden of adverse childhood experiences in England and Wales: A combined primary data study of five surveys. BMJ Open, 10(6), e036374. 10.1136/bmjopen-2019-036374 PMC7282338 32513892

[bibr45-15248380251358223] Hunt M. Auriemma J. Cashaw A. C. A. (2003). Self-report bias and underreporting of depression on the BDI-II. Journal of Personality Assessment, 80(1), 26–30. 10.1207/S15327752JPA8001_10 12584064

[bibr46-15248380251358223] Kalmakis K. A. Chandler G. E. (2015). Health consequences of adverse childhood experiences: A systematic review. Journal of the American Association of Nurse Practitioners, 27(8), 457–465. 10.1002/2327-6924.12215 25755161

[bibr47-15248380251358223] Karcher N. R. Niendam T. A. Barch D. M. (2020). Adverse childhood experiences and psychotic-like experiences are associated above and beyond shared correlates: Findings from the adolescent brain cognitive development study. Schizophrenia Research, 222, 235–242. 10.1016/j.schres.2020.05.045 32522466 PMC7572890

[bibr48-15248380251358223] Kisely S. Abajobir A. A. Mills R. Strathearn L. Clavarino A. Najman J. M. (2018). Child maltreatment and mental health problems in adulthood: Birth cohort study. The British Journal of Psychiatry, 213(6), 698–703. 10.1192/bjp.2018.207 30475193

[bibr49-15248380251358223] Lacey R. E. Minnis H. (2020). Practitioner review: Twenty years of research with adverse childhood experience scores—Advantages, disadvantages and applications to practice. Journal of Child Psychology and Psychiatry, 61(2), 116–130. 10.1111/jcpp.13135 31609471

[bibr50-15248380251358223] Le Strat Y. Dubertret C. Le Foll B . (2011). Child marriage in the United States and its association with mental health in women. Pediatrics, 128(3), 524–530. 10.1542/peds.2011-0961 21873691

[bibr51-15248380251358223] LeMasters K. Bates L. M. Chung E. O. Gallis J. A. Hagaman A. Scherer E. Sikander S. Staley B. S. Zalla L. C. Zivich P. N. Maselko J. (2021). Adverse childhood experiences and depression among women in rural Pakistan. BMC Public Health, 21(1), 400. 10.1186/s12889-021-10409-4 33632175 PMC7905421

[bibr52-15248380251358223] Lereya S. T. Copeland W. E. Zammit S. Wolke D. (2015). Bully/victims: A longitudinal, population-based cohort study of their mental health. European Child & Adolescent Psychiatry, 24(12), 1461–1471. 10.1007/s00787-015-0705-5 25825225

[bibr53-15248380251358223] Llabre M. M. Hadi F. (2009). War-related exposure and psychological distress as predictors of health and sleep: A longitudinal study of Kuwaiti children. Psychosomatic Medicine, 71(7), 776–783. 10.1097/PSY.0b013e3181ae6aee 19592513

[bibr54-15248380251358223] Macaskill P. Walter S. D. Irwig L. (2001). A comparison of methods to detect publication bias in meta-analysis. Statistics in Medicine, 20(4), 641–654. 10.1002/sim.698 11223905

[bibr55-15248380251358223] Masiano S. P. Yu X. Tembo T. Wetzel E. Mphande M. Khama I. Mkandawire A. Chitani M. Liwimbi O. Udedi M. Mazenga A. Nyasulu P. Abrams E. Ahmed S. Kim M. H. (2022). The relationship between adverse childhood experiences and common mental disorders among pregnant women living with HIV in Malawi. Journal of Affective Disorders, 312, 159–168. 10.1016/j.jad.2022.06.028 35752220 PMC9892657

[bibr56-15248380251358223] Masten A. S. Cicchetti D. (2010). Developmental cascades. Development and Psychopathology, 22(3), 491–495. 10.1017/S0954579410000222 20576173

[bibr57-15248380251358223] McManus S. Gunnell D. Cooper C. Bebbington P. E. Howard L. M. Brugha T. Jenkins R. Hassiotis A. Weich S. Appleby L. (2019). Prevalence of non-suicidal self-harm and service contact in England, 2000-14: Repeated cross-sectional surveys of the general population. The Lancet. Psychiatry, 6(7), 573–581. 10.1016/S2215-0366(19)30188-9 31175059 PMC7646286

[bibr58-15248380251358223] Meinck F. Neelakantan L. Steele B. Jochim J. Davies L. M. Boyes M. Barlow J. Dunne M. (2023). Measuring violence against children: A COSMIN systematic review of the psychometric properties of child and adolescent self-report measures. Trauma, Violence, & Abuse, 24(3), 1832–1847. 10.1177/15248380221082152 PMC10240621 35446727

[bibr59-15248380251358223] Mittendorfer-Rutz E. Rasmussen F. Lange T. (2012). A life-course study on effects of parental markers of morbidity and mortality on offspring’s suicide attempt. PLoS One, 7(12), e51585. 10.1371/journal.pone.0051585 PMC3520946 23251584

[bibr60-15248380251358223] Nadan Y. Spilsbury J. C. Korbin J. E. (2015). Culture and context in understanding child maltreatment: Contributions of intersectionality and neighborhood-based research. Child Abuse Neglect, 41. 10.1016/j.chiabu.2014.10.021 25466427

[bibr61-15248380251358223] Nakagawa S. Lagisz M. Jennions M. D. Koricheva J. Noble D. W. A. Parker T. H. Sánchez-Tójar A. Yang Y. O’Dea R. E. (2022). Methods for testing publication bias in ecological and evolutionary meta-analyses. Methods in Ecology and Evolution, 13(1), 4–21. 10.1111/2041-210X.13724

[bibr62-15248380251358223] Negriff S. (2020). ACEs are not equal: Examining the relative impact of household dysfunction versus childhood maltreatment on mental health in adolescence. Social Science and Medicine, 245, 112696. 10.1016/j.socscimed.2019.112696 31785426 PMC6961803

[bibr63-15248380251358223] Newbury J. B. Arseneault L. Moffitt T. E. Caspi A. Danese A. Baldwin J. R. Fisher H. L. (2018). Measuring childhood maltreatment to predict early-adult psychopathology: Comparison of prospective informant-reports and retrospective self-reports. Journal of Psychiatric Research, 96, 57–64. 10.1016/j.jpsychires.2017.09.020 28965006 PMC5725307

[bibr64-15248380251358223] Nikulina V. Widom C. S. Czaja S. (2011). The role of childhood neglect and childhood poverty in predicting mental health, academic achievement and crime in adulthood. American Journal of Community Psychology, 48(3), 309–321. 10.1007/s10464-010-9385-y 21116706 PMC7197378

[bibr65-15248380251358223] Norman R. E. Byambaa M. De R. Butchart A. Scott J. Vos T. (2012). The long-term health consequences of child physical abuse, emotional abuse, and neglect: A systematic review and meta-analysis. PLoS Medicine, 9(11), e1001349. 10.1371/journal.pmed.1001349 PMC3507962 23209385

[bibr66-15248380251358223] Oláh B. Fekete Z. Kuritárné Szabó I. Kovács-Tóth B. (2023). Validity and reliability of the 10-item Adverse Childhood Experiences Questionnaire (ACE-10) among adolescents in the child welfare system. Frontiers in Public Health, 11. 10.3389/fpubh.2023.1258798 PMC10691263 38045975

[bibr67-15248380251358223] Ottisova L. Smith P. Shetty H. Stahl D. Downs J. Oram S. (2018). Psychological consequences of child trafficking: A historical cohort study of trafficked children in contact with secondary mental health services. PLoS One, 13(3), e0192321. 10.1371/journal.pone.0192321 PMC5843209 29518168

[bibr68-15248380251358223] Page M. J. McKenzie J. E. Bossuyt P. M. Boutron I. Hoffmann T. C. Mulrow C. D. Shamseer L. Tetzlaff J. M. Akl E. A. Brennan S. E. Chou R. Glanville J. Grimshaw J. M. Hróbjartsson A. Lalu M. M. Li T. Loder E. W. Mayo-Wilson E. McDonald S. . . . Moher D. (2021). The PRISMA 2020 statement: An updated guideline for reporting systematic reviews. BMJ, 372, n71. 10.1136/bmj.n71 PMC8005924 33782057

[bibr69-15248380251358223] Peters J. L. Sutton A. J. Jones D. R. Abrams K. R. Rushton L. (2006). Comparison of two methods to detect publication bias in meta-analysis. JAMA, 295(6), 676–680. 10.1001/jama.295.6.676 16467236

[bibr70-15248380251358223] Peters J. L. Sutton A. J. Jones D. R. Abrams K. R. Rushton L. (2007). Performance of the trim and fill method in the presence of publication bias and between-study heterogeneity. Statistics in Medicine, 26(25), 4544–4562. 10.1002/sim.2889 17476644

[bibr71-15248380251358223] Pinto R. Correia L. Maia Â . (2014). Assessing the reliability of retrospective reports of adverse childhood experiences among adolescents with documented childhood maltreatment. Journal of Family Violence, 29(4), 431–438. 10.1007/s10896-014-9602-9

[bibr72-15248380251358223] Poole J. C. Dobson K. S. Pusch D. (2017). Anxiety among adults with a history of childhood adversity: Psychological resilience moderates the indirect effect of emotion dysregulation. Journal of Affective Disorders, 217, 144–152. 10.1016/j.jad.2017.03.047 28410477

[bibr73-15248380251358223] Ramiro L. S. Madrid B. J. Brown D. W. (2010). Adverse childhood experiences (ACE) and health-risk behaviors among adults in a developing country setting. Child Abuse & Neglect, 34(11), Article 11. 10.1016/j.chiabu.2010.02.012 20888640

[bibr74-15248380251358223] Reuben A. Moffitt T. E. Caspi A. Belsky D. W. Harrington H. Schroeder F. Hogan S. Ramrakha S. Poulton R. Danese A. (2016). Lest we forget: Comparing retrospective and prospective assessments of adverse childhood experiences in the prediction of adult health. Journal of Child Psychology and Psychiatry, 57(10), 1103–1112. 10.1111/jcpp.12621 27647050 PMC5234278

[bibr75-15248380251358223] Rodgers M. A. Pustejovsky J. E. (2021). Evaluating meta-analytic methods to detect selective reporting in the presence of dependent effect sizes. Psychological Methods, 26(2), 141–160. 10.1037/met0000300 32673040

[bibr76-15248380251358223] Sacks V. Murphy D. (2018). The prevalence of adverse childhood experiences, nationally, by state, and by race or ethnicity. Child Trends. https://www.childtrends.org/publications/prevalence-adverse-childhood-experiences-nationally-state-race-ethnicity

[bibr77-15248380251358223] Sahle B. W. Reavley N. J. Li W. Morgan A. J. Yap M. B. H. Reupert A. Jorm A. F. (2022). The association between adverse childhood experiences and common mental disorders and suicidality: An umbrella review of systematic reviews and meta-analyses. European Child & Adolescent Psychiatry, 31(10), 1489–1499. 10.1007/s00787-021-01745-2 33638709

[bibr78-15248380251358223] Salo M. Appleton A. A. Tracy M. (2021). Childhood adversity trajectories and violent behaviors in adolescence and early adulthood. Journal of Interpersonal Violence, 08862605211006366. 10.1177/08862605211006366 PMC8521560 33858246

[bibr79-15248380251358223] Schalinski I. Teicher M. H. Nischk D. Hinderer E. Müller O. Rockstroh B. (2016). Type and timing of adverse childhood experiences differentially affect severity of PTSD, dissociative and depressive symptoms in adult inpatients. BMC Psychiatry, 16(1), 295. 10.1186/s12888-016-1004-5 27543114 PMC4992284

[bibr80-15248380251358223] Sedgwick P. (2013). Meta-analyses: How to read a funnel plot. BMJ, 346, f1342. 10.1136/bmj.f1342 26377337

[bibr81-15248380251358223] Soares A. L. G. Howe L. D. Matijasevich A. Wehrmeister F. C. Menezes A. M. B. Gonçalves H. (2016). Adverse childhood experiences: Prevalence and related factors in adolescents of a Brazilian birth cohort. Child Abuse & Neglect, 51, 21–30. 10.1016/j.chiabu.2015.11.017 26707919 PMC4710615

[bibr82-15248380251358223] Stang A. (2010). Critical evaluation of the Newcastle-Ottawa scale for the assessment of the quality of nonrandomized studies in meta-analyses. European Journal of Epidemiology, 25(9), 603–605. 10.1007/s10654-010-9491-z 20652370

[bibr83-15248380251358223] Stanley T. D. Doucouliagos H. (2014). Meta-regression approximations to reduce publication selection bias. Research Synthesis Methods, 5(1), 60–78. 10.1002/jrsm.1095 26054026

[bibr84-15248380251358223] Stansfeld S. A. Clark C. Smuk M. Power C. Davidson T. Rodgers B. (2017). Childhood adversity and midlife suicidal ideation. Psychological Medicine, 47(2), 327–340. 10.1017/S0033291716002336 27762177 PMC5216460

[bibr85-15248380251358223] Stapinski L. A. Bowes L. Wolke D. Pearson R. M. Mahedy L. Button K. S. Lewis G. Araya R. (2014). Peer victimization during adolescence and risk for anxiety disorders in adulthood: A prospective cohort study. Depression and Anxiety, 31(7), 574–582. 10.1002/da.22270 24788688 PMC4190687

[bibr86-15248380251358223] Sterne J. A. C. Gavaghan D. Egger M. (2000). Publication and related bias in meta-analysis: Power of statistical tests and prevalence in the literature. Journal of Clinical Epidemiology, 53(11), 1119–1129. 10.1016/S0895-4356(00)00242-0 11106885

[bibr87-15248380251358223] Sterne J. A. C. Harbord R. (2004). Funnel plots in meta-analysis. Stata Journal, 4(2), 127–141.

[bibr88-15248380251358223] Terrin N. Schmid C. H. Lau J. Olkin I. (2003). Adjusting for publication bias in the presence of heterogeneity. Statistics in Medicine, 22(13), 2113–2126. 10.1002/sim.1461 12820277

[bibr89-15248380251358223] The United Nations Convention on the Rights of the Child [UNCRC]. (2019). The United Nations Convention on the Rights of the Child. https://downloads.unicef.org.uk/wp-content/uploads/2010/05/UNCRC_united_nations_convention_on_the_rights_of_the_child.pdf

[bibr90-15248380251358223] Thurston C. Murray A. L. Franchino-Olsen H. Meinck F. (2023). Prospective longitudinal associations between adverse childhood experiences and adult mental health outcomes: A protocol for a systematic review and meta-analysis. Systematic Reviews, 12(1), 181. 10.1186/s13643-023-02330-1 37777785 PMC10541707

[bibr91-15248380251358223] USC-Office of Population Studies Foundation, Inc. (OPS). (2019). USC-Office of Population Studies Foundation, Inc. (OPS). https://www.opsusc.org/paper_series.php

[bibr92-15248380251358223] van Dam D. S. van der Ven E. Velthorst E. Selten J. P. Morgan C. de Haan L . (2012). Childhood bullying and the association with psychosis in non-clinical and clinical samples: A review and meta-analysis. Psychological Medicine, 42(12), 2463–2474. 10.1017/S0033291712000360 22400714

[bibr93-15248380251358223] van den Noortgate W. López-López J. A. Marín-Martínez F. Sánchez-Meca J . (2015). Meta-analysis of multiple outcomes: A multilevel approach. Behavior Research Methods, 47(4), 1274–1294. 10.3758/s13428-014-0527-2 25361866

[bibr94-15248380251358223] van Os J. Linscott R. J. Myin-Germeys I. Delespaul P. Krabbendam L. (2009). A systematic review and meta-analysis of the psychosis continuum: Evidence for a psychosis proneness-persistence-impairment model of psychotic disorder. Psychological Medicine, 39(2), 179–195. 10.1017/S0033291708003814 18606047

[bibr95-15248380251358223] Vaswani N. (2019). Bullying behaviours: Adverse experiences for all involved? Centre for Youth & Criminal Justice. https://www.cycj.org.uk/wp-content/uploads/2019/06/Bullying-adverse-experiences.pdf

[bibr96-15248380251358223] Victora C. G. Barros F. C. (2012). Cohorts in low- and middle-income countries: From still photographs to full-length movies. The Journal of Adolescent Health, 51(6), S3–S4. 10.1016/j.jadohealth.2012.09.003 PMC3508418 23283157

[bibr97-15248380251358223] Viechtbauer W. (2010). Conducting meta-analyses in R with the metafor package. Journal of Statistical Software, 36(3), 1–48. 10.18637/jss.v036.i03

[bibr98-15248380251358223] Viechtbauer W. (2015). Package ‘metafor’. https://citeseerx.ist.psu.edu/document?repid=rep1&x0026;type=pdf&x0026;doi=d8c6d24678982bdfcf1bf051cf503d9f991fd6fa

[bibr99-15248380251358223] von Hippel P. T . (2015). The heterogeneity statistic I^2^ can be biased in small meta-analyses. BMC Medical Research Methodology, 15(1), 35. 10.1186/s12874-015-0024-z 25880989 PMC4410499

[bibr100-15248380251358223] Wells G. Shea B. O’Connell D. Peterson J. Welch V. Losos M. Tugwell P. (2000). The Newcastle-Ottawa scale (NOS) for assessing the quality of nonrandomised studies in meta-analyses. https://www.ohri.ca/programs/clinical_epidemiology/oxford.asp

[bibr101-15248380251358223] Westermair A. L. Stoll A. M. Greggersen W. Kahl K. G. Hüppe M. Schweiger U. (2018). All unhappy childhoods are unhappy in their own way-differential impact of dimensions of adverse childhood experiences on adult mental health and health behavior. Frontiers in Psychiatry, 9, 198. 10.3389/fpsyt.2018.00198 29875707 PMC5974933

[bibr102-15248380251358223] Wilson D. B. (n.d.). Practical meta-analysis effect size calculator [Online calculator]. Campbell Collaboration. https://www.campbellcollaboration.org/research-resources/effect-size-calculator.html

[bibr103-15248380251358223] Wolke D. Lereya S. T. Fisher H. L. Lewis G. Zammit S. (2014). Bullying in elementary school and psychotic experiences at 18 years: A longitudinal, population-based cohort study. Psychological Medicine, 44(10), 2199–2211. 10.1017/S0033291713002912 24342773

