# Supplementary material for: Prospective Longitudinal Associations Between Adverse Childhood Experiences and Adult Mental Health Outcomes: Systematic Review and Meta-Analysis
Source: Trauma Violence Abuse. 2025 Aug 17;27(4):1700–18. doi: 10.1177/15248380251358223 (PMC13578656; doi:10.1177/15248380251358223)
Supplement: Supplementary file 1 [file sj-docx-1-tva-10.1177_15248380251358223.docx]

**Appendix A**

**Inclusion Criteria Table**

Inclusion Criteria using the PEO model where applicable.

| **Criteria** | **Inclusion** |
| --- | --- |
| Design | There must have been at least 2 points; one in which prospective ACE(s) measured during childhood (< 18 years old) and one in which mental health outcome(s) were measured in adulthood (>17 years). |
| Publication Status | Studies were peer-reviewed or non-peer-reviewed. |
| Location | The study was conducted in any geographical location. |
| Source of Evidence | Findings were from books, research articles, government documents, conference abstracts, annual reports, dissertations, and theses. |
| Date | The studies were published from 1990- 2021, but the data may have been collected before 1990. This specific period was chosen as it aligns with the drafting of the United Nations Convention on the Rights of the Child by the United Nations (UNCRC) (UNCRC, 2019). |
| Language | The studies were readily available in English due to limited research resources prohibiting translation. |
| **P**opulation | The samples were from the general human population but did not need to be nationally representative. |
| **P**opulation | The sample did not need to be selected based on mental ill-health or exposure to ACEs. However, the sample could have been “clinical” or “special” in the sense that they may have been recruited through an outpatient clinic or attend an outpatient clinic (for example, an outpatient clinic for depression or an outpatient HIV clinic). They may also have had a diagnosed mental illness or be at a higher risk of experiencing ACEs or poor mental health through, for example, coming from a disadvantaged background. |
| **E**xposure | The study could include prospective measures of ACEs obtained through official reports of child maltreatment (such as police, court, or child welfare records) that were recorded when the adult participant was a child and were then incorporated into the study through data linkage. |
| **E**xposure | The ACE(s) was/were reported by anyone who knew the child (I.e., teacher, parent, caregiver, child themselves) or child welfare records or court records. |
| **O**utcomes | Adult mental health was measured using a continuous or cut-off score approach. Clinical records of adult mental health such as patient records were also included. |
| **O**utcomes | The studies did not need to use clinical diagnostic tools for ascertaining mental health conditions but included measurement(s) of mental health outcomes were validated measures of PTSD, depression, anxiety, suicidal ideation, self-harm, or psychotic-like-experiences. The tool's validation did not need to be in the context the study was set in. |

**Appendix B**

**Exclusion Criteria Table**

Exclusion Criteria using the PEO model where applicable.

| **Criteria** | **Exclusion** |
| --- | --- |
| Design | Cross-sectional studies. |
| Design | Analyses of interventions were excluded. |
| Design | Research containing non-empirical work, qualitative research, and non-original data such as commentaries. |
| Date | Studies published before 1990. |
| **P**opulation | Whilst certain “clinical” samples were included (see inclusion criteria), specific groups that live outside of the general community such as inpatients in psychiatric hospitals or those currently imprisoned were excluded. This was to allow for more meaningful effect size comparisons between studies as effects in such sample may be overinflated. |
| **E**xposure | Any study that only included retrospective measures of ACEs (I.e., the study only includes the reporting of ACEs when the subjects are 18 and over) was excluded. |
| **E**xposure | Studies that only used crude measurements of adversity such as “were you abused as a child” without asking about specific events (such as being hit for physical abuse). |
| **E**xposure | Studies in which the only adversity was a physical trauma without a maltreatment/ abuse/household dysfunction or chronic component (e.g., a car crash). |
| **O**utcomes | Studies that only used crude measurements of mental health such as “have you ever been depressed” without asking about specific symptoms (such as hopelessness for depressed mood) |

**Appendix C**

**Ovid Search Strategy Example**

**SEARCH 1- Embase (1980 to 2021 Week 19); Global Health (1973 to 2021 Week 19); APA PsycInfo (1806 to 2021 Week 19); and Ovid MEDLINE® and Epub Ahead of Print, In-Process, In-Data-Review & Other Non-Indexed Citations and Daily (1946 to 2021 Week 19).**

10-June-21

| **Domain** | **String #** | **Search Term** | **Limiters Applied** | **Results** |
| --- | --- | --- | --- | --- |
| POPULATION  (Children) | 1 | (Child* OR Infan* OR Teen* OR Adolescen* OR School-age* OR "School Age*" OR Toddler* OR Baby OR Babies OR Newborn* OR Kid* OR Minor* OR Preschool* OR Pre-School* OR Underage* OR Under-age* OR Juvenile* OR Perinatal* OR Youth* OR "Young Pe*").m_titl. |  | 3936707 |
|  | 2 | #1 | Humans [Limit not valid in Global Health or APA Psyc  Info]  Yr = “1990- Current” | 2864040 |
| INTEREST  (Adverse Childhood Experiences: excluding parental adversities) | 3 | (Advers* OR ACE* OR "Adverse Childhood Experience*" OR Trauma* OR Violence OR Abuse* OR Terrori* OR Coup* OR Riot* OR Revolution OR "Household Dysfunction*" OR Neglect* OR Bully* OR Bullie* OR Conflict* OR Exploit* OR Polyvictimi* OR Victimi* OR "Peer Violence" OR Peer-Violence OR Marriage OR War* OR Assault* OR Prostitut* OR Sex-Work* OR "Sex Work*" OR Traffick* OR Displace*).m_titl. |  | 1847836 |
|  | 4 | #3 | Humans [Limit not valid in Global Health or APA PsycInfo]  Yr = “1990- Current” | 1104923 |
| INTEREST  (Adverse Childhood Experiences: exclusively parental adversities) | 5 | (Parent* adj1 (Substance* or Alcohol* or "Illicit Drug*" or "Prescription Drug*" or Drug* or Cannabis or Cocaine or Heroin or Separat* or Divorce* or Break* or Mental* or Disorder* or Death or Morbidit* or Ill*)).m_titl. |  | 6701 |
|  | 6 | #5 | Humans [Limit not valid in Global Health or APA PsycInfo]  Yr = “1990- Current” | 5071 |
| OUTCOME  (Adult Mental Health) | 7 | (“Mental Health” OR "Mental* Ill*" OR "Mental Disorder*" OR Anxiety OR "Panic Disorder" OR "Obsessive Compulsive Disorder" OR "Social Phobia" OR "Post-Traumatic Stress" OR "Post Traumatic Stress" OR "Acute Stress" OR Suicid* OR Self-Harm* OR Self-Injur* OR Self-Poison* OR Self-Punish* OR "Self Harm*" OR "Self Injur*" OR "Self Poison*" OR "Self Punish*" OR Psycho* OR Schizo* OR Delusion* OR Hallucination* OR Paranoi* OR "Magical Thinking" OR Abnormal* OR Paranormal* OR "Odd Beliefs*” OR Depress*).m_titl. |  | 2342919 |
|  | 8 | #7 | Humans [Limit not valid in Global Health or APA PsycInfo]  Yr = “1990- Current” | 1690597 |
| DESIGN | 9 | (Longitudinal* or Cohort* or Panel* or Prospective*).mp. [mp=ti, ab, hw, tn, ot, dm, mf, dv, kw, fx, dq, tc, id, tm, mh, sh, bt, cc, nm, kf, ox, px, rx, an, ui, sy] |  | 555223 |
|  | 10 | #9 | Humans [Limit not valid in Global Health or APA Psyc  Info]  Yr = “1990- Current” |  |
| ALL DOMAINS | 11 | 2 AND (4 OR 6) AND 8 AND 10 |  | 4176 |
| ALL DOMAINS (DE-DUPLICATED) |  |  |  | 1193 |

**Appendix D**

**Full Reference List of the 62 Studies Included in the Final Review**

Abajobir, A. A., Kisely, S., Scott, J. G., Williams, G., Clavarino, A., Strathearn, L., & Najman, J. M. (2017). Childhood Maltreatment and Young Adulthood Hallucinations, Delusional Experiences, and Psychosis: A Longitudinal Study. *Schizophrenia Bulletin*, *43*(5), 1045–1055. <https://doi.org/10.1093/schbul/sbw175>

Ackard, D. M., Eisenberg, M. E., & Neumark-Sztainer, D. (2007). Long-Term Impact of Adolescent Dating Violence on the Behavioral and Psychological Health of Male and Female Youth. *The Journal of Pediatrics*, *151*(5), 476–481. <https://doi.org/10.1016/j.jpeds.2007.04.034>

Akbulut-Yuksel, M., Tekin, E., & Turan, B. (2022). *World War II Blues: The Long–lasting Mental Health Effect of Childhood Trauma* (Working Paper 30284). National Bureau of Economic Research. <https://doi.org/10.3386/w30284>

Alaie, I., Låftman, S. B., Jonsson, U., & Bohman, H. (2020). Parent–youth conflict as a predictor of depression in adulthood: A 15-year follow-up of a community-based cohort. *European Child & Adolescent Psychiatry*, *29*(4), 527–536. <https://doi.org/10.1007/s00787-019-01368-8>

Björkenstam, E., Hjern, A., Björkenstam, C., & Kosidou, K. (2018). Association of Cumulative Childhood Adversity and Adolescent Violent Offending With Suicide in Early Adulthood. *JAMA Psychiatry*, *75*(2), 185–193. <https://doi.org/10.1001/jamapsychiatry.2017.3788>

Björkenstam, E., Vinnerljung, B., & Hjern, A. (2017). Impact of childhood adversities on depression in early adulthood: A longitudinal cohort study of 478,141 individuals in Sweden. *Journal of Affective Disorders*, *223*, 95–100. <https://doi.org/10.1016/j.jad.2017.07.030>

Boden, J. M., Stockum, S. van, Horwood, L. J., & Fergusson, D. M. (2016). Bullying victimization in adolescence and psychotic symptomatology in adulthood: Evidence from a 35-year study. *Psychological Medicine*, *46*(6), 1311–1320. <https://doi.org/10.1017/S0033291715002962>

Bohman, H., Låftman, S. B., Päären, A., & Jonsson, U. (2017). Parental separation in childhood as a risk factor for depression in adulthood: A community-based study of adolescents screened for depression and followed up after 15 years. *BMC Psychiatry*, *17*(1), 117. <https://doi.org/10.1186/s12888-017-1252-z>

Buchanan, A., & Ten Brinke, J. (1998). Measuring outcomes for children: Early parenting experiences, conflict, maladjustment, and depression in adulthood. *Children and Youth Services Review*, *20*(3), 251–278. <https://doi.org/10.1016/S0190-7409(98)00006-1>

Cadmus-Romm, D. (2004). *Emotional abuse and neglect in adolescence as predictors of psychosocial adjustment in young adulthood—ProQuest*. <https://www.proquest.com/openview/bc9deb206e763f1295cf3dad9f3f7d7a/1?pq-origsite=gscholar&cbl=18750&diss=y>

Chen, W.-Y., Corvo, K., Lee, Y., & Hahm, H. C. (2017). Longitudinal Trajectory of Adolescent Exposure to Community Violence and Depressive Symptoms Among Adolescents and Young Adults: Understanding the Effect of Mental Health Service Usage. *Community Mental Health Journal*, *53*(1), 39–52. <https://doi.org/10.1007/s10597-016-0031-5>

Choe, D. E., Stoddard, S. A., & Zimmerman, M. A. (2014). Developmental trajectories of African American adolescents’ family conflict: Differences in mental health problems in young adulthood. *Developmental Psychology*, *50*(4), 1226–1232. <https://doi.org/10.1037/a0035199>

Clark, C., Caldwell, T., Power, C., & Stansfeld, S. A. (2010). Does the Influence of Childhood Adversity on Psychopathology Persist Across the Lifecourse? A 45-Year Prospective Epidemiologic Study. *Annals of Epidemiology*, *20*(5), 385–394. <https://doi.org/10.1016/j.annepidem.2010.02.008>

Copeland, W. E., Shanahan, L., Hinesley, J., Chan, R. F., Aberg, K. A., Fairbank, J. A., van den Oord, E. J. C. G., & Costello, E. J. (2018). Association of Childhood Trauma Exposure With Adult Psychiatric Disorders and Functional Outcomes. *JAMA Network Open*, *1*(7), e184493. <https://doi.org/10.1001/jamanetworkopen.2018.4493>

Copeland, W. E., Wolke, D., Angold, A., & Costello, E. J. (2013). Adult Psychiatric Outcomes of Bullying and Being Bullied by Peers in Childhood and Adolescence. *JAMA Psychiatry*, *70*(4), 419–426. <https://doi.org/10.1001/jamapsychiatry.2013.504>

Cutajar, M. C., Mullen, P. E., Ogloff, J. R. P., Thomas, S. D., Wells, D. L., & Spataro, J. (2010). Psychopathology in a large cohort of sexually abused children followed up to 43 years. *Child Abuse & Neglect*, *34*(11), 813–822. <https://doi.org/10.1016/j.chiabu.2010.04.004>

Dantchev, S., Hickman, M., Heron, J., Zammit, S., & Wolke, D. (2019). The Independent and Cumulative Effects of Sibling and Peer Bullying in Childhood on Depression, Anxiety, Suicidal Ideation, and Self-Harm in Adulthood. *Frontiers in Psychiatry*, *10*. <https://doi.org/10.3389/fpsyt.2019.00651>

Dubowitz, H., Roesch, S., Lewis, T., Thompson, R., English, D., & Kotch, J. B. (2022). Neglect in Childhood, Problem Behavior in Adulthood. *Journal of Interpersonal Violence*, *37*(23–24), NP22047–NP22065. <https://doi.org/10.1177/08862605211067008>

Eitle, D. (2022). Examining Direct and Indirect Pathways between Adolescent Extra-Familial Criminal Victimization and Adult Mental Health. *American Journal of Criminal Justice*, *47*(5), 941–959. <https://doi.org/10.1007/s12103-021-09615-y>

Gallo, E. A. G., De Mola, C. L., Wehrmeister, F., Gonçalves, H., Kieling, C., & Murray, J. (2017). Childhood maltreatment preceding depressive disorder at age 18 years: A prospective Brazilian birth cohort study. *Journal of Affective Disorders*, *217*, 218–224. <https://doi.org/10.1016/j.jad.2017.03.065>

Geoffroy, M.-C., Arseneault, L., Girard, A., Ouellet-Morin, I., & Power, C. (2023). Association of childhood bullying victimisation with suicide deaths: Findings from a 50-year nationwide cohort study. *Psychological Medicine*, *53*(9), 4152–4159. <https://doi.org/10.1017/S0033291722000836>

Gondek, D., Patalay, P., & Lacey, R. E. (2021). Adverse childhood experiences and multiple mental health outcomes through adulthood: A prospective birth cohort study. *SSM - Mental Health*, *1*, 100013. <https://doi.org/10.1016/j.ssmmh.2021.100013>

Heinze, J. E., Cook, S. H., Wood, E. P., Dumadag, A. C., & Zimmerman, M. A. (2018). Friendship Attachment Style Moderates the Effect of Adolescent Exposure to Violence on Emerging Adult Depression and Anxiety Trajectories. *Journal of Youth and Adolescence*, *47*(1), 177–193. <https://doi.org/10.1007/s10964-017-0729-x>

Herrenkohl, T. I., Hong, S., Klika, J. B., Herrenkohl, R. C., & Russo, M. J. (2013). Developmental Impacts of Child Abuse and Neglect Related to Adult Mental Health, Substance Use, and Physical Health. *Journal of Family Violence*, *28*(2), 191–199. <https://doi.org/10.1007/s10896-012-9474-9>

Herrenkohl, T. I., Kosterman, R., Hawkins, J. D., & Mason, W. A. (2009). Effects of Growth in Family Conflict in Adolescence on Adult Depressive Symptoms: Mediating and Moderating Effects of Stress and School Bonding. *Journal of Adolescent Health*, *44*(2), 146–152. <https://doi.org/10.1016/j.jadohealth.2008.07.005>

Hjern, A., Palacios, J., & Vinnerljung, B. (2023). Early childhood adversity and non-affective psychosis: A study of refugees and international adoptees in Sweden. *Psychological Medicine*, *53*(5), 1914–1923. <https://doi.org/10.1017/S003329172100355X>

Hoprekstad, Ø. L., Hetland, J., Wold, B., Torp, H., & Valvatne Einarsen, S. (2021). Exposure to Bullying Behaviors at Work and Depressive Tendencies: The Moderating Role of Victimization From Bullying During Adolescence. *Journal of Interpersonal Violence*, *36*(23–24), 11576–11601. <https://doi.org/10.1177/0886260519900272>

Horan, J. M., & Widom, C. S. (2015). Does Age of Onset of Risk Behaviors Mediate the Relationship Between Child Abuse and Neglect and Outcomes in Middle Adulthood? *Journal of Youth and Adolescence*, *44*(3), 670–682. <https://doi.org/10.1007/s10964-014-0161-4>

Huurre, T., Junkkari, H., & Aro, H. (2006). Long–term Psychosocial effects of parental divorce. *European Archives of Psychiatry and Clinical Neuroscience*, *256*(4), 256–263. <https://doi.org/10.1007/s00406-006-0641-y>

Iob, E., Baldwin, J. R., Plomin, R., & Steptoe, A. (2021). Adverse childhood experiences, daytime salivary cortisol, and depressive symptoms in early adulthood: A longitudinal genetically informed twin study. *Translational Psychiatry*, *11*(1), 1–10. <https://doi.org/10.1038/s41398-021-01538-w>

Kisely, S., Abajobir, A. A., Mills, R., Strathearn, L., Clavarino, A., & Najman, J. M. (2018). Child maltreatment and mental health problems in adulthood: Birth cohort study. *The British Journal of Psychiatry*, *213*(6), 698–703. <https://doi.org/10.1192/bjp.2018.207>

Kisely, S., Strathearn, L., Mills, R., & Najman, J. M. (2021). A comparison of the psychological outcomes of self-reported and agency-notified child abuse in a population-based birth cohort at 30-year-follow-up. *Journal of Affective Disorders*, *280*, 167–172. <https://doi.org/10.1016/j.jad.2020.11.017>

Kisely, S., Strathearn, L., & Najman, J. M. (2022). A comparison of psychosis-like symptoms following self-reported and agency-notified child abuse in a population-based birth cohort at 30-year-follow-up. *Schizophrenia Research*, *239*, 116–122. <https://doi.org/10.1016/j.schres.2021.11.029>

Klomek, A. B., Sourander, A., Kumpulainen, K., Piha, J., Tamminen, T., Moilanen, I., Almqvist, F., & Gould, M. S. (2008). Childhood bullying as a risk for later depression and suicidal ideation among Finnish males. *Journal of Affective Disorders*, *109*(1), 47–55. <https://doi.org/10.1016/j.jad.2007.12.226>

Koenen, K. C., Moffitt, T. E., Poulton, R., Martin, J., & Caspi, A. (2007). Early childhood factors associated with the development of post-traumatic stress disorder: Results from a longitudinal birth cohort. *Psychological Medicine*, *37*(2), 181–192. <https://doi.org/10.1017/S0033291706009019>

Lee, C., Cronley, C., White, H. R., Mun, E.-Y., Stouthamer-Loeber, M., & Loeber, R. (2012). Racial Differences in the Consequences of Childhood Maltreatment for Adolescent and Young Adult Depression, Heavy Drinking, and Violence. *Journal of Adolescent Health*, *50*(5), 443–449. <https://doi.org/10.1016/j.jadohealth.2011.09.014>

Lereya, S. T., Copeland, W. E., Costello, E. J., & Wolke, D. (2015). Adult mental health consequences of peer bullying and maltreatment in childhood: Two cohorts in two countries. *The Lancet Psychiatry*, *2*(6), 524–531. <https://doi.org/10.1016/S2215-0366(15)00165-0>

Lereya, S. T., Copeland, W. E., Zammit, S., & Wolke, D. (2015). Bully/victims: A longitudinal, population-based cohort study of their mental health. *European Child & Adolescent Psychiatry*, *24*(12), 1461–1471. <https://doi.org/10.1007/s00787-015-0705-5>

Liu, Y., Heron, J., Hickman, M., Zammit, S., & Wolke, D. (2022). Prenatal stress and offspring depression in adulthood: The mediating role of childhood trauma. *Journal of Affective Disorders*, *297*, 45–52. <https://doi.org/10.1016/j.jad.2021.10.019>

Moffitt, T. E., Caspi, A., Harrington, H., Milne, B. J., Melchior, M., Goldberg, D., & Poulton, R. (2007). Generalized anxiety disorder and depression: Childhood risk factors in a birth cohort followed to age 32. *Psychological Medicine*, *37*(3), 441–452. <https://doi.org/10.1017/S0033291706009640>

Moretti, M. M., & Craig, S. G. (2013). Maternal versus paternal physical and emotional abuse, affect regulation and risk for depression from adolescence to early adulthood. *Child Abuse & Neglect*, *37*(1), 4–13. <https://doi.org/10.1016/j.chiabu.2012.09.015>

Naicker, S. N., Norris, S. A., & Richter, L. M. (2021). Secondary analysis of retrospective and prospective reports of adverse childhood experiences and mental health in young adulthood: Filtered through recent stressors. *eClinicalMedicine*, *40*. <https://doi.org/10.1016/j.eclinm.2021.101094>

Palosaari, U., Aro, H., & Laippala, P. (1996). Parental divorce and depression in young adulthood: Adolescents’ closeness to parents and self-esteem as mediating factor. *Acta Psychiatrica Scandinavica*, *93*(1), 20–26. <https://doi.org/10.1111/j.1600-0447.1996.tb10614.x>

Ramirez, D., & Haas, S. A. (2021). The Long Arm of Conflict: How Timing Shapes the Impact of Childhood Exposure to War. *Demography*, *58*(3), 951–974. <https://doi.org/10.1215/00703370-9114715>

Richter, L. M., Mathews, S., Nonterah, E., & Masilela, L. (2018). A longitudinal perspective on boys as victims of childhood sexual abuse in South Africa: Consequences for adult mental health. *Child Abuse & Neglect*, *84*, 1–10. <https://doi.org/10.1016/j.chiabu.2018.07.016>

Shanahan, L., Copeland, W. E., Costello, E. J., & Angold, A. (2011). Child-, adolescent- and young adult-onset depressions: Differential risk factors in development? *Psychological Medicine*, *41*(11), 2265–2274. <https://doi.org/10.1017/S0033291711000675>

Sigurdson, J. F., Undheim, A. M., Wallander, J. L., Lydersen, S., & Sund, A. M. (2015). The long-term effects of being bullied or a bully in adolescence on externalizing and internalizing mental health problems in adulthood. *Child and Adolescent Psychiatry and Mental Health*, *9*(1), 42. <https://doi.org/10.1186/s13034-015-0075-2>

Sourander, A., Jensen, P., Rönning, J. A., Niemelä, S., Helenius, H., Sillanmäki, L., Kumpulainen, K., Piha, J., Tamminen, T., Moilanen, I., & Almqvist, F. (2007). What Is the Early Adulthood Outcome of Boys Who Bully or Are Bullied in Childhood? The Finnish “From a Boy to a Man” Study. *Pediatrics*, *120*(2), 397–404. <https://doi.org/10.1542/peds.2006-2704>

Sousa, C., Mason, W. A., Herrenkohl, T. I., Prince, D., Herrenkohl, R. C., & Russo, M. J. (2018). Direct and indirect effects of child abuse and environmental stress: A lifecourse perspective on adversity and depressive symptoms. *American Journal of Orthopsychiatry*, *88*(2), 180–188. <https://doi.org/10.1037/ort0000283>

Spatz Widom, C., DuMont, K., & Czaja, S. J. (2007). A Prospective Investigation of Major Depressive Disorder and Comorbidity in Abused and Neglected Children Grown Up. *Archives of General Psychiatry*, *64*(1), 49–56. <https://doi.org/10.1001/archpsyc.64.1.49>

Stansfeld, S. A., Clark, C., Smuk, M., Power, C., Davidson, T., & Rodgers, B. (2017). Childhood adversity and midlife suicidal ideation. *Psychological Medicine*, *47*(2), 327–340. <https://doi.org/10.1017/S0033291716002336>

Stapinski, L. A., Bowes, L., Wolke, D., Pearson, R. M., Mahedy, L., Button, K. S., Lewis, G., & Araya, R. (2014). Peer Victimization During Adolescence and Risk for Anxiety Disorders in Adulthood: A Prospective Cohort Study. *Depression and Anxiety*, *31*(7), 574–582. <https://doi.org/10.1002/da.22270>

Størksen, I., Røysamb, E., Moum, T., & Tambs, K. (2005). Adolescents with a childhood experience of parental divorce: A longitudinal study of mental health and adjustment. *Journal of Adolescence*, *28*(6), 725–739. <https://doi.org/10.1016/j.adolescence.2005.01.001>

Takizawa, R., Maughan, B., & Arseneault, L. (2014). Adult Health Outcomes of Childhood Bullying Victimization: Evidence From a Five-Decade Longitudinal British Birth Cohort. *American Journal of Psychiatry*, *171*(7), 777–784. <https://doi.org/10.1176/appi.ajp.2014.13101401>

Thompson, M. P., & Kingree, J. B. (2022). Adverse childhood experiences, sexual victimization, and suicide ideation and attempts: A longitudinal path analysis spanning 22 years. *American Journal of Orthopsychiatry*, *92*(3), 302–309. <https://doi.org/10.1037/ort0000613>

van Dulmen, M. H. M., Klipfel, K. M., Mata, A. D., Schinka, K. C., Claxton, S. E., Swahn, M. H., & Bossarte, R. M. (2012). Cross-Lagged Effects Between Intimate Partner Violence Victimization and Suicidality From Adolescence Into Adulthood. *Journal of Adolescent Health*, *51*(5), 510–516. <https://doi.org/10.1016/j.jadohealth.2012.02.015>

Vassallo, S., Edwards, B., Renda, J., & Olsson, C. A. (2014). Bullying in Early Adolescence and Antisocial Behavior and Depression Six Years Later: What Are the Protective Factors? *Journal of School Violence*, *13*(1), 100–124. <https://doi.org/10.1080/15388220.2013.840643>

Widom, C. S. (1999). Posttraumatic Stress Disorder in Abused and Neglected Children Grown Up. *American Journal of Psychiatry*, *156*(8), 1223–1229. <https://doi.org/10.1176/ajp.156.8.1223>

Winding, T. N., Skouenborg, L. A., Mortensen, V. L., & Andersen, J. H. (2020). Is bullying in adolescence associated with the development of depressive symptoms in adulthood?: A longitudinal cohort study. *BMC Psychology*, *8*(1), 122. <https://doi.org/10.1186/s40359-020-00491-5>

Wong, W. H., Kuo, W.-H., Sobolewski, C., Bhatia, I., & Ip, P. (2020). The Association Between Child Abuse and Attempted Suicide: A Retrospective Cohort Study. *Crisis*, *41*(3), 196–204. <https://doi.org/10.1027/0227-5910/a000625>

Zhu, X., Griffiths, H., Eisner, M., Hepp, U., Ribeaud, D., & Murray, A. L. (2022a). Developmental associations between bullying victimization and suicidal ideation and direct self-injurious behavior in adolescence and emerging adulthood. *Journal of Child Psychology and Psychiatry*, *63*(7), 820–828. <https://doi.org/10.1111/jcpp.13529>

Zhu, X., Griffiths, H., Eisner, M., Hepp, U., Ribeaud, D., & Murray, A. L. (2022b). Developmental Relations Between Bullying Victimization and Suicidal Ideation in Middle Adolescence and Emerging Adulthood: Do Internalizing Problems and Substance Use Mediate Their Links? *Journal of Youth and Adolescence*, *51*(9), 1745–1759. <https://doi.org/10.1007/s10964-022-01630-4>

**Appendix E**

**Summary of Study Characteristics for the Final Studies Exploring Prospective Associations between Adverse Childhood Experiences and Adult Mental Ill-Health (*N* = 62)**

| Characteristic | | N Studies (% to nearest whole number) | |  |
| --- | --- | --- | --- | --- |
| **Year Published** | |  | |  |
|  | 1990-1994 | | 0 (0) | |
|  | 1995-1999 | | 3 (5) | |
|  | 2000-2004 | | 1 (5) | |
|  | 2005-2009 | | 10 (16) | |
|  | 2010—2014 | | 12 (19) | |
|  | 2015-2019 | | 18 (29) | |
|  | 2020-2023 | | 18 (29) | |
| **Study Format** | |  | |  |
|  | Article | | 61 (98) | |
|  | Dissertation/ Thesis | | 1 (2) | |
| **Country**  **(n.b., a study by Lereya et al. (2015) used 2 cohorts from the UK and USA that were both counted as 1 study for each country)** | |  | |  |
|  | USA | | 20 (31) | |
|  | UK or Great Britain | | 12 (19) | |
|  | Australia | | 6 (10) | |
|  | Sweden | | 5 (8) | |
|  | Finland | | 4 (6) | |
|  | New Zealand | | 3 (5) | |
|  | Norway | | 3 (5) | |
|  | South Africa  Switzerland | | 2 (3)  2(3) | |
|  | Germany  Canada | | 1(2)  1 (2) | |
|  | Denmark | | 1 (2) | |
|  | Brazil | | 1 (2) | |
|  | Kuwait  Europe | | 1 (2)  1 (2) | |
| **Country Income Level** | |  | |  |
|  | High | | 59 (95) | |
|  | Upper middle | | 3 (5) | |
|  | Lower middle | | 0 (0) | |
|  | Low | | 0 (0) | |
| **Sample size**  **(n.b., the study by Lereya et al. (2015) used 2 cohorts with different sample sizes that were both counted as 0.5 studies for each sample size)** | |  | |  |
|  | 0-499 | | 9 (15) | |
|  | 500-999 | | 9 (15) | |
|  | 1000-1999 | | 15.5 (25) | |
|  | 2000-2999 | | 6 (10) | |
|  | 3000-3999 | | 5 (8) | |
|  | 4000-4999 | | 2.5 (4) | |
|  | 5000-5999 | | 1 (2) | |
|  | 6000-6999 | | 0 (0) | |
|  | 7000-7999 | | 2 (3) | |
|  | 8000-8999 | | 1 (2) | |
|  | 9000-9999 | | 2 (3) | |
|  | 10000-10999 | | 2(3) | |
|  | 11000-11999  14,000-14,999  28,000-28,999  40,000-44,999 | | 1 (2)  1 (2)  1 (2)  1 (2) | |
|  | 400000-499999  Unclear | | 2 (3)  1 (2) | |
| **Sample source/cohort**  **(n.b., the study by Lereya et al. (2015) used 2 separate sample sources that were both counted as 0.5 studies for each source)** | |  | |  |
|  | Unnamed cohort (USA *N*= 5, Sweden *N*= 3, Finland *N*= 2, Canada *N*= 1, Kuwait *N*= 1, Australia *N*= 1, Germany *N*=1, Europe-wide *N=*1) | | 15 (24) | |
|  | The 1958 British Birth Cohort study – GB | | 6 (8) | |
|  | The Avon Longitudinal Study of Parents and Children (ALSPAC) – UK | | 4.5 (7) | |
|  | The Great Smoky Mountain Study – USA | | 3.5 (7) | |
|  | The National Longitudinal Study of Adolescent to Adult Health (Add Health) – USA | | 4 (6) | |
|  | Mater-University of Queensland Study  of Pregnancy (MUSP) – Australia  The Zurich Project on the Social Development from Childhood to Adulthood (z-proso) | | 4 (6)  2 (3) | |
|  | Uppsala Longitudinal Adolescent Depression Study – Sweden | | 2 (4) | |
|  | From a Boy to a Man- Finland | | 2 (4) | |
|  | Birth to Twenty Plus Cohort (BT20+) – South Africa | | 2 (4) | |
|  | Dunedin Multidisciplinary Health and Development Study – New Zealand | | 2 (4) | |
|  | Lehigh Longitudinal Study, which began in 1973 – 1974 -USA | | 2 (4) | |
|  | The Norwegian Longitudinal Health Behavior Study (NLHBS) - Norway | | 1 (2) | |
|  | Seattle Social Development Project (SSDP) -USA | | 1 (2) | |
|  | the Pittsburgh Youth Study (PYS) - USA | | 1 (2) | |
|  | Project EAT – USA | | 1 (2) | |
|  | The Nord-Trøndelag Health study (HUNT) – Norway | | 1 (2) | |
|  | Christchurch Health and Development Study (CHDS) – New Zealand | | 1 (2) | |
|  | Petersen Adolescent Mental Health Study 1978-1990 – USA | | 1 (2) | |
|  | The Australian Temperament Project (ATP) – Australia | | 1 (2) | |
|  | The 1993 Pelotas birth cohort – Brazil | | 1 (2) | |
|  | West Jutland Cohort Study (VestLiv) – Denmark | | 1 (2) | |
|  | The Youth and Mental Health Study – Norway  The Twins Early Development Study  Longitudinal Studies of Child Abuse and Neglect (LONGSCAN) | | 1 (2)  1 (2)  1 (2) | |
| **ACE type**  **(n.b., multiple studies measured more than 1 ACE and thus the total studies summary equals more than 62 studies)** | |  | |  |
|  | Bullying Victimization | | 17 (27) | |
|  | Neglect | | 12 (19) | |
|  | Sexual Abuse | | 10 (16) | |
|  | Non-specific Child Abuse/ Child maltreatment | | 9 (15) | |
|  | Parental Separation | | 9 (15) | |
|  | Parental Mental Illness (including measures of mother’s internalizing symptoms) | | 8 (13) | |
|  | Physical Abuse | | 7(11) | |
|  | Conflict with parents/caregiver/family including DV | | 7 (11) | |
|  | Cumulative Exposure to Abuse/ Maltreatment/ Violence (e.g., “multiple abuse”) | | 6 (10) | |
|  | Bullying Perpetration and Victimization (note this was included as there was a victimization component but bully perpetration alone was not included as an ACE) | | 6 (10) | |
|  | Conflict with parents/caregiver/family including DV | | 7 (11) | |
|  | Parental Non-specific Loss (including things such as absence, separation, illegitimacy, in care or death) | | 6 (10) | |
|  | Parental Death | | 6 (10) | |
|  | Emotional Abuse | | 6 (10) | |
|  | Non-specific Child Abuse and Neglect Combined Measure | | 4 (6) | |
|  | Parental Substance Misuse | | 4 (6) | |
|  | Intimate Partner Violence (note this was included in retrospect as the studies definitions of IPV matches characteristics of child abuse (physical, emotional, or sexual abuse) | | 3 (5) | |
|  | In care (note this was included in retrospect as the studies definitions of being in care matches characteristics of parental loss | | 3 (5) | |
|  | Non-specific violence events (such as death of family member through violence) | | 3 (5) | |
|  | Parental Criminality/ Imprisonment | | 3 (5) | |
|  | Household Morbidity incl. mental illness | | 3 (4) | |
|  | Collective Violence | | 3 (4) | |
|  | Community Violence | | 2 (3) | |
|  | Child Welfare Intervention | | 2 (3) | |
|  | Child Displacement | | 1(2) | |
|  | Non-specific loss events (such as death of family member or best friend/ parental divorce) | | 1 (2) | |
|  | Household Death | | 1 (2) | |
| **Sources of ACEs reporting** | |  | |  |
|  | Self-reported through written self-reports or structured interview | | 28 (45) | |
|  | Official records/registers of Adverse Childhood Experiences (e.g., court records, police records, national registers, medical databases, patient records) | | 15 (24) | |
|  | Self-reported and informant reported | | 10 (16) | |
|  | Informant-reported (parent/ caregiver/ teacher) | | 3 (5) | |
|  | A mixture of self-reported, informant reported and objective professional-reported (e.g., observations of neglected appearance) | | 3 (5) | |
|  | Maternal reports and unclear for certain adversities  Official records and self-reported | | 2 (3)  1(2) | |
| **ACEs Measurement Tool** | |  | |  |
|  | Custom-made self/informant-report scale | | 21 (36) | |
|  | An existing self/informant-report scale | | 15 (24) | |
|  | Official records (e.g., court records, police records, national registers, medical databases, patient records) | | 16 (22) | |
|  | Mixture of existing scale and custom-made items | | 5 (8) | |
|  | Unclear | | 3 (6) | |
|  | Mixture of custom-made items and objective medical examination | | 2 (4) | |
| **Mental health Outcomes**  **(n.b., multiple studies measured more than 1 outcome and thus the total studies in this descriptive summary will equal more than 50 studies/ 100%).** | |  | |  |
|  | Depression | | 48 (77) | |
|  | Anxiety | | 21 (34) | |
|  | Suicidality | | 12 (19) | |
|  | Post-Traumatic Stress Disorder | | 7 (11) | |
|  | Psychotic-Like Experiences | | 7 (11) | |
|  | Self-harm | | 1 (2) (please note SH outcomes were lifetime measures only and thus had to be excluded) | |
| **Outcome Measurement Tool** | |  | |  |
|  | Validated scale used prospectively | | 50 (81) | |
|  | Official medical records/registers that use official diagnoses | | 6 (10) | |
|  | Custom items that capture official diagnostic symptoms | | 5 (8) | |
|  | Validated scale used prospectively and Custom items that capture official diagnostic symptoms | | 1(2) | |
| **Analytic Method**  **(n.b., this is a summary of the analytic methods relevant to the data included in this review only and numerous studies contained a variety of other analytic methods)** | |  | |  |
|  | Logistic Regression | | 36 (62) | |
|  | Correlational analysis | | 7 (14) | |
|  | Poisson Regression | | 3.5 (6) | |
|  | Linear Regression | | 3.5 (4) | |
|  | Means and Standard Deviations | | 2 (4) | |
|  | Logistic Regression and Poisson Regression | | 2 (4) | |
|  | Cox Hazard Models | | 2 (2) | |
|  | Chi Square Analysis and Logistic Regression | | 1 (2) | |
|  | 2x2 Frequency table  Random Intercept Cross-Lagged Panel Models | | 1 (2)  2(2) | |
| Latent Class Analysis 1(2)  Difference In Differences Regression 1(2) | | | |  |

**Appendix F**

**Total Star Ratings for Each Study (Out of a Possible 8 Stars) from the Newcastle-Ottawa Scale (NOS) for Assessing the Quality of Nonrandomized Studies in Meta-Analyses**

| **Study** | **Total Star Rating** |
| --- | --- |
| Abajobir, A. A., Kisely, S., Scott, J. G., Williams, G., Clavarino, A., Strathearn, L., & Najman, J. M. (2017). Childhood Maltreatment and Young Adulthood Hallucinations, Delusional Experiences, and Psychosis: A Longitudinal Study. *Schizophrenia Bulletin*, *43*(5), 1045–1055. <https://doi.org/10.1093/schbul/sbw175> | 6 |
| Ackard, D. M., Eisenberg, M. E., & Neumark-Sztainer, D. (2007). Long-Term Impact of Adolescent Dating Violence on the Behavioral and Psychological Health of Male and Female Youth. *The Journal of Pediatrics*, *151*(5), 476–481. <https://doi.org/10.1016/j.jpeds.2007.04.034> | 6 |
| Akbulut-Yuksel, M., Tekin, E., & Turan, B. (2022). *World War II Blues: The Long–lasting Mental Health Effect of Childhood Trauma* (Working Paper 30284). National Bureau of Economic Research. <https://doi.org/10.3386/w30284> | 6 |
| Alaie, I., Låftman, S. B., Jonsson, U., & Bohman, H. (2020). Parent–youth conflict as a predictor of depression in adulthood: A 15-year follow-up of a community-based cohort. *European Child & Adolescent Psychiatry*, *29*(4), 527–536. <https://doi.org/10.1007/s00787-019-01368-8> | 7 |
| Björkenstam, E., Hjern, A., Björkenstam, C., & Kosidou, K. (2018). Association of Cumulative Childhood Adversity and Adolescent Violent Offending With Suicide in Early Adulthood. *JAMA Psychiatry*, *75*(2), 185–193. <https://doi.org/10.1001/jamapsychiatry.2017.3788> | 6 |
| Björkenstam, E., Vinnerljung, B., & Hjern, A. (2017). Impact of childhood adversities on depression in early adulthood: A longitudinal cohort study of 478,141 individuals in Sweden. *Journal of Affective Disorders*, *223*, 95–100. <https://doi.org/10.1016/j.jad.2017.07.030> | 6 |
| Boden, J. M., Stockum, S. van, Horwood, L. J., & Fergusson, D. M. (2016). Bullying victimization in adolescence and psychotic symptomatology in adulthood: Evidence from a 35-year study. *Psychological Medicine*, *46*(6), 1311–1320. <https://doi.org/10.1017/S0033291715002962> | 7 |
| Bohman, H., Låftman, S. B., Päären, A., & Jonsson, U. (2017). Parental separation in childhood as a risk factor for depression in adulthood: A community-based study of adolescents screened for depression and followed up after 15 years. *BMC Psychiatry*, *17*(1), 117. <https://doi.org/10.1186/s12888-017-1252-z> | 6 |
| Buchanan, A., & Ten Brinke, J. (1998). Measuring outcomes for children: Early parenting experiences, conflict, maladjustment, and depression in adulthood. *Children and Youth Services Review*, *20*(3), 251–278. <https://doi.org/10.1016/S0190-7409(98)00006-1> | 5 |
| Cadmus-Romm, D. (2004). *Emotional abuse and neglect in adolescence as predictors of psychosocial adjustment in young adulthood—ProQuest*. <https://www.proquest.com/openview/bc9deb206e763f1295cf3dad9f3f7d7a/1?pq-origsite=gscholar&cbl=18750&diss=y> | 5 |
| Chen, W.-Y., Corvo, K., Lee, Y., & Hahm, H. C. (2017). Longitudinal Trajectory of Adolescent Exposure to Community Violence and Depressive Symptoms Among Adolescents and Young Adults: Understanding the Effect of Mental Health Service Usage. *Community Mental Health Journal*, *53*(1), 39–52. <https://doi.org/10.1007/s10597-016-0031-5> | 5 |
| Choe, D. E., Stoddard, S. A., & Zimmerman, M. A. (2014). Developmental trajectories of African American adolescents’ family conflict: Differences in mental health problems in young adulthood. *Developmental Psychology*, *50*(4), 1226–1232. <https://doi.org/10.1037/a0035199> | 6 |
| Clark, C., Caldwell, T., Power, C., & Stansfeld, S. A. (2010). Does the Influence of Childhood Adversity on Psychopathology Persist Across the Lifecourse? A 45-Year Prospective Epidemiologic Study. *Annals of Epidemiology*, *20*(5), 385–394. <https://doi.org/10.1016/j.annepidem.2010.02.008> | 5 |
| Copeland, W. E., Shanahan, L., Hinesley, J., Chan, R. F., Aberg, K. A., Fairbank, J. A., van den Oord, E. J. C. G., & Costello, E. J. (2018). Association of Childhood Trauma Exposure With Adult Psychiatric Disorders and Functional Outcomes. *JAMA Network Open*, *1*(7), e184493. <https://doi.org/10.1001/jamanetworkopen.2018.4493> | 7 |
| Copeland, W. E., Wolke, D., Angold, A., & Costello, E. J. (2013). Adult Psychiatric Outcomes of Bullying and Being Bullied by Peers in Childhood and Adolescence. *JAMA Psychiatry*, *70*(4), 419–426. <https://doi.org/10.1001/jamapsychiatry.2013.504> | 6 |
| Cutajar, M. C., Mullen, P. E., Ogloff, J. R. P., Thomas, S. D., Wells, D. L., & Spataro, J. (2010). Psychopathology in a large cohort of sexually abused children followed up to 43 years. *Child Abuse & Neglect*, *34*(11), 813–822. <https://doi.org/10.1016/j.chiabu.2010.04.004> | 7 |
| Dantchev, S., Hickman, M., Heron, J., Zammit, S., & Wolke, D. (2019). The Independent and Cumulative Effects of Sibling and Peer Bullying in Childhood on Depression, Anxiety, Suicidal Ideation, and Self-Harm in Adulthood. *Frontiers in Psychiatry*, *10*. <https://doi.org/10.3389/fpsyt.2019.00651> | 5 |
| Dubowitz, H., Roesch, S., Lewis, T., Thompson, R., English, D., & Kotch, J. B. (2022). Neglect in Childhood, Problem Behavior in Adulthood. *Journal of Interpersonal Violence*, *37*(23–24), NP22047–NP22065. <https://doi.org/10.1177/08862605211067008> | 6 |
| Eitle, D. (2022). Examining Direct and Indirect Pathways between Adolescent Extra-Familial Criminal Victimization and Adult Mental Health. *American Journal of Criminal Justice*, *47*(5), 941–959. <https://doi.org/10.1007/s12103-021-09615-y> | 6 |
| Gallo, E. A. G., De Mola, C. L., Wehrmeister, F., Gonçalves, H., Kieling, C., & Murray, J. (2017). Childhood maltreatment preceding depressive disorder at age 18 years: A prospective Brazilian birth cohort study. *Journal of Affective Disorders*, *217*, 218–224. <https://doi.org/10.1016/j.jad.2017.03.065> | 5 |
| Geoffroy, M.-C., Arseneault, L., Girard, A., Ouellet-Morin, I., & Power, C. (2023). Association of childhood bullying victimisation with suicide deaths: Findings from a 50-year nationwide cohort study. *Psychological Medicine*, *53*(9), 4152–4159. <https://doi.org/10.1017/S0033291722000836> | 6 |
| Gondek, D., Patalay, P., & Lacey, R. E. (2021). Adverse childhood experiences and multiple mental health outcomes through adulthood: A prospective birth cohort study. *SSM - Mental Health*, *1*, 100013. <https://doi.org/10.1016/j.ssmmh.2021.100013> | 5 |
| Heinze, J. E., Cook, S. H., Wood, E. P., Dumadag, A. C., & Zimmerman, M. A. (2018). Friendship Attachment Style Moderates the Effect of Adolescent Exposure to Violence on Emerging Adult Depression and Anxiety Trajectories. *Journal of Youth and Adolescence*, *47*(1), 177–193. <https://doi.org/10.1007/s10964-017-0729-x> | 6 |
| Herrenkohl, T. I., Hong, S., Klika, J. B., Herrenkohl, R. C., & Russo, M. J. (2013). Developmental Impacts of Child Abuse and Neglect Related to Adult Mental Health, Substance Use, and Physical Health. *Journal of Family Violence*, *28*(2), 191–199. <https://doi.org/10.1007/s10896-012-9474-9> | 6 |
| Herrenkohl, T. I., Kosterman, R., Hawkins, J. D., & Mason, W. A. (2009). Effects of Growth in Family Conflict in Adolescence on Adult Depressive Symptoms: Mediating and Moderating Effects of Stress and School Bonding. *Journal of Adolescent Health*, *44*(2), 146–152. <https://doi.org/10.1016/j.jadohealth.2008.07.005> | 5 |
| Hjern, A., Palacios, J., & Vinnerljung, B. (2023). Early childhood adversity and non-affective psychosis: A study of refugees and international adoptees in Sweden. *Psychological Medicine*, *53*(5), 1914–1923. <https://doi.org/10.1017/S003329172100355X> | 7 |
| Hoprekstad, Ø. L., Hetland, J., Wold, B., Torp, H., & Valvatne Einarsen, S. (2021). Exposure to Bullying Behaviors at Work and Depressive Tendencies: The Moderating Role of Victimization From Bullying During Adolescence. *Journal of Interpersonal Violence*, *36*(23–24), 11576–11601. <https://doi.org/10.1177/0886260519900272> | 6 |
| Horan, J. M., & Widom, C. S. (2015). Does Age of Onset of Risk Behaviors Mediate the Relationship Between Child Abuse and Neglect and Outcomes in Middle Adulthood? *Journal of Youth and Adolescence*, *44*(3), 670–682. <https://doi.org/10.1007/s10964-014-0161-4> | 6 |
| Huurre, T., Junkkari, H., & Aro, H. (2006). Long–term Psychosocial effects of parental divorce. *European Archives of Psychiatry and Clinical Neuroscience*, *256*(4), 256–263. <https://doi.org/10.1007/s00406-006-0641-y> | 6 |
| Iob, E., Baldwin, J. R., Plomin, R., & Steptoe, A. (2021). Adverse childhood experiences, daytime salivary cortisol, and depressive symptoms in early adulthood: A longitudinal genetically informed twin study. *Translational Psychiatry*, *11*(1), 1–10. <https://doi.org/10.1038/s41398-021-01538-w> | 5 |
| Kisely, S., Abajobir, A. A., Mills, R., Strathearn, L., Clavarino, A., & Najman, J. M. (2018). Child maltreatment and mental health problems in adulthood: Birth cohort study. *The British Journal of Psychiatry*, *213*(6), 698–703. <https://doi.org/10.1192/bjp.2018.207> | 6 |
| Kisely, S., Strathearn, L., Mills, R., & Najman, J. M. (2021). A comparison of the psychological outcomes of self-reported and agency-notified child abuse in a population-based birth cohort at 30-year-follow-up. *Journal of Affective Disorders*, *280*, 167–172. <https://doi.org/10.1016/j.jad.2020.11.017> | 6 |
| Kisely, S., Strathearn, L., & Najman, J. M. (2022). A comparison of psychosis-like symptoms following self-reported and agency-notified child abuse in a population-based birth cohort at 30-year-follow-up. *Schizophrenia Research*, *239*, 116–122. <https://doi.org/10.1016/j.schres.2021.11.029> | 6 |
| Klomek, A. B., Sourander, A., Kumpulainen, K., Piha, J., Tamminen, T., Moilanen, I., Almqvist, F., & Gould, M. S. (2008). Childhood bullying as a risk for later depression and suicidal ideation among Finnish males. *Journal of Affective Disorders*, *109*(1), 47–55. <https://doi.org/10.1016/j.jad.2007.12.226> | 6 |
| Koenen, K. C., Moffitt, T. E., Poulton, R., Martin, J., & Caspi, A. (2007). Early childhood factors associated with the development of post-traumatic stress disorder: Results from a longitudinal birth cohort. *Psychological Medicine*, *37*(2), 181–192. <https://doi.org/10.1017/S0033291706009019> | 6 |
| Lee, C., Cronley, C., White, H. R., Mun, E.-Y., Stouthamer-Loeber, M., & Loeber, R. (2012). Racial Differences in the Consequences of Childhood Maltreatment for Adolescent and Young Adult Depression, Heavy Drinking, and Violence. *Journal of Adolescent Health*, *50*(5), 443–449. <https://doi.org/10.1016/j.jadohealth.2011.09.014> | 6 |
| Lereya, S. T., Copeland, W. E., Costello, E. J., & Wolke, D. (2015). Adult mental health consequences of peer bullying and maltreatment in childhood: Two cohorts in two countries. *The Lancet Psychiatry*, *2*(6), 524–531. <https://doi.org/10.1016/S2215-0366(15)00165-0> | 6 |
| Lereya, S. T., Copeland, W. E., Zammit, S., & Wolke, D. (2015). Bully/victims: A longitudinal, population-based cohort study of their mental health. *European Child & Adolescent Psychiatry*, *24*(12), 1461–1471. <https://doi.org/10.1007/s00787-015-0705-5> | 7 |
| Liu, Y., Heron, J., Hickman, M., Zammit, S., & Wolke, D. (2022). Prenatal stress and offspring depression in adulthood: The mediating role of childhood trauma. *Journal of Affective Disorders*, *297*, 45–52. <https://doi.org/10.1016/j.jad.2021.10.019> | 5 |
| Moffitt, T. E., Caspi, A., Harrington, H., Milne, B. J., Melchior, M., Goldberg, D., & Poulton, R. (2007). Generalized anxiety disorder and depression: Childhood risk factors in a birth cohort followed to age 32. *Psychological Medicine*, *37*(3), 441–452. <https://doi.org/10.1017/S0033291706009640> | 6 |
| Moretti, M. M., & Craig, S. G. (2013). Maternal versus paternal physical and emotional abuse, affect regulation and risk for depression from adolescence to early adulthood. *Child Abuse & Neglect*, *37*(1), 4–13. <https://doi.org/10.1016/j.chiabu.2012.09.015> | 6 |
| Naicker, S. N., Norris, S. A., & Richter, L. M. (2021). Secondary analysis of retrospective and prospective reports of adverse childhood experiences and mental health in young adulthood: Filtered through recent stressors. *eClinicalMedicine*, *40*. <https://doi.org/10.1016/j.eclinm.2021.101094> | 5 |
| Palosaari, U., Aro, H., & Laippala, P. (1996). Parental divorce and depression in young adulthood: Adolescents’ closeness to parents and self-esteem as mediating factor. *Acta Psychiatrica Scandinavica*, *93*(1), 20–26. <https://doi.org/10.1111/j.1600-0447.1996.tb10614.x> | 4 |
| Ramirez, D., & Haas, S. A. (2021). The Long Arm of Conflict: How Timing Shapes the Impact of Childhood Exposure to War. *Demography*, *58*(3), 951–974. <https://doi.org/10.1215/00703370-9114715> | 6 |
| Richter, L. M., Mathews, S., Nonterah, E., & Masilela, L. (2018). A longitudinal perspective on boys as victims of childhood sexual abuse in South Africa: Consequences for adult mental health. *Child Abuse & Neglect*, *84*, 1–10. <https://doi.org/10.1016/j.chiabu.2018.07.016> | 6 |
| Shanahan, L., Copeland, W. E., Costello, E. J., & Angold, A. (2011). Child-, adolescent- and young adult-onset depressions: Differential risk factors in development? *Psychological Medicine*, *41*(11), 2265–2274. <https://doi.org/10.1017/S0033291711000675> | 5 |
| Sigurdson, J. F., Undheim, A. M., Wallander, J. L., Lydersen, S., & Sund, A. M. (2015). The long-term effects of being bullied or a bully in adolescence on externalizing and internalizing mental health problems in adulthood. *Child and Adolescent Psychiatry and Mental Health*, *9*(1), 42. <https://doi.org/10.1186/s13034-015-0075-2> | 5 |
| Sourander, A., Jensen, P., Rönning, J. A., Niemelä, S., Helenius, H., Sillanmäki, L., Kumpulainen, K., Piha, J., Tamminen, T., Moilanen, I., & Almqvist, F. (2007). What Is the Early Adulthood Outcome of Boys Who Bully or Are Bullied in Childhood? The Finnish “From a Boy to a Man” Study. *Pediatrics*, *120*(2), 397–404. <https://doi.org/10.1542/peds.2006-2704> | 6 |
| Sousa, C., Mason, W. A., Herrenkohl, T. I., Prince, D., Herrenkohl, R. C., & Russo, M. J. (2018). Direct and indirect effects of child abuse and environmental stress: A lifecourse perspective on adversity and depressive symptoms. *American Journal of Orthopsychiatry*, *88*(2), 180–188. <https://doi.org/10.1037/ort0000283> | 6 |
| Spatz Widom, C., DuMont, K., & Czaja, S. J. (2007). A Prospective Investigation of Major Depressive Disorder and Comorbidity in Abused and Neglected Children Grown Up. *Archives of General Psychiatry*, *64*(1), 49–56. <https://doi.org/10.1001/archpsyc.64.1.49> | 6 |
| Stansfeld, S. A., Clark, C., Smuk, M., Power, C., Davidson, T., & Rodgers, B. (2017). Childhood adversity and midlife suicidal ideation. *Psychological Medicine*, *47*(2), 327–340. <https://doi.org/10.1017/S0033291716002336> | 6 |
| Stapinski, L. A., Bowes, L., Wolke, D., Pearson, R. M., Mahedy, L., Button, K. S., Lewis, G., & Araya, R. (2014). Peer Victimization During Adolescence and Risk for Anxiety Disorders in Adulthood: A Prospective Cohort Study. *Depression and Anxiety*, *31*(7), 574–582. <https://doi.org/10.1002/da.22270> | 7 |
| Størksen, I., Røysamb, E., Moum, T., & Tambs, K. (2005). Adolescents with a childhood experience of parental divorce: A longitudinal study of mental health and adjustment. *Journal of Adolescence*, *28*(6), 725–739. <https://doi.org/10.1016/j.adolescence.2005.01.001> | 5 |
| Takizawa, R., Maughan, B., & Arseneault, L. (2014). Adult Health Outcomes of Childhood Bullying Victimization: Evidence From a Five-Decade Longitudinal British Birth Cohort. *American Journal of Psychiatry*, *171*(7), 777–784. <https://doi.org/10.1176/appi.ajp.2014.13101401> | 6 |
| Thompson, M. P., & Kingree, J. B. (2022). Adverse childhood experiences, sexual victimization, and suicide ideation and attempts: A longitudinal path analysis spanning 22 years. *American Journal of Orthopsychiatry*, *92*(3), 302–309. <https://doi.org/10.1037/ort0000613> | 5 |
| van Dulmen, M. H. M., Klipfel, K. M., Mata, A. D., Schinka, K. C., Claxton, S. E., Swahn, M. H., & Bossarte, R. M. (2012). Cross-Lagged Effects Between Intimate Partner Violence Victimization and Suicidality From Adolescence Into Adulthood. *Journal of Adolescent Health*, *51*(5), 510–516. <https://doi.org/10.1016/j.jadohealth.2012.02.015> | 6 |
| Vassallo, S., Edwards, B., Renda, J., & Olsson, C. A. (2014). Bullying in Early Adolescence and Antisocial Behavior and Depression Six Years Later: What Are the Protective Factors? *Journal of School Violence*, *13*(1), 100–124. <https://doi.org/10.1080/15388220.2013.840643> | 5 |
| Widom, C. S. (1999). Posttraumatic Stress Disorder in Abused and Neglected Children Grown Up. *American Journal of Psychiatry*, *156*(8), 1223–1229. <https://doi.org/10.1176/ajp.156.8.1223> | 5 |
| Winding, T. N., Skouenborg, L. A., Mortensen, V. L., & Andersen, J. H. (2020). Is bullying in adolescence associated with the development of depressive symptoms in adulthood?: A longitudinal cohort study. *BMC Psychology*, *8*(1), 122. <https://doi.org/10.1186/s40359-020-00491-5> | 5 |
| Wong, W. H., Kuo, W.-H., Sobolewski, C., Bhatia, I., & Ip, P. (2020). The Association Between Child Abuse and Attempted Suicide: A Retrospective Cohort Study. *Crisis*, *41*(3), 196–204. <https://doi.org/10.1027/0227-5910/a000625> | 8 |
| Zhu, X., Griffiths, H., Eisner, M., Hepp, U., Ribeaud, D., & Murray, A. L. (2022a). Developmental associations between bullying victimization and suicidal ideation and direct self-injurious behavior in adolescence and emerging adulthood. *Journal of Child Psychology and Psychiatry*, *63*(7), 820–828. <https://doi.org/10.1111/jcpp.13529> | 5 |
| Zhu, X., Griffiths, H., Eisner, M., Hepp, U., Ribeaud, D., & Murray, A. L. (2022b). Developmental Relations Between Bullying Victimization and Suicidal Ideation in Middle Adolescence and Emerging Adulthood: Do Internalizing Problems and Substance Use Mediate Their Links? *Journal of Youth and Adolescence*, *51*(9), 1745–1759. <https://doi.org/10.1007/s10964-022-01630-4> | 55 5 |

**Appendix G**

**List of Conversions to Odds Ratios and Reasons for Lack of Conversion Where Applicable**

| **Study** | **Conversion Method** |
| --- | --- |
| Akbulut-Yuksel, M., Tekin, E., & Turan, B. (2022). *World War II Blues: The Long–lasting Mental Health Effect of Childhood Trauma* (Working Paper 30284). National Bureau of Economic Research. <https://doi.org/10.3386/w30284> | Insufficient information |
| Björkenstam, E., Hjern, A., Björkenstam, C., & Kosidou, K. (2018). Association of Cumulative Childhood Adversity and Adolescent Violent Offending With Suicide in Early Adulthood. *JAMA Psychiatry*, *75*(2), 185–193. <https://doi.org/10.1001/jamapsychiatry.2017.3788> | Not comparable - incidence rate ratios |
| Björkenstam, E., Vinnerljung, B., & Hjern, A. (2017). Impact of childhood adversities on depression in early adulthood: A longitudinal cohort study of 478,141 individuals in Sweden. *Journal of Affective Disorders*, *223*, 95–100. <https://doi.org/10.1016/j.jad.2017.07.030> | Not comparable – hazard ratios |
| Boden, J. M., Stockum, S. van, Horwood, L. J., & Fergusson, D. M. (2016). Bullying victimization in adolescence and psychotic symptomatology in adulthood: Evidence from a 35-year study. *Psychological Medicine*, *46*(6), 1311–1320. <https://doi.org/10.1017/S0033291715002962> | Means and standard deviations |
| Buchanan, A., & Ten Brinke, J. (1998). Measuring outcomes for children: Early parenting experiences, conflict, maladjustment, and depression in adulthood. *Children and Youth Services Review*, *20*(3), 251–278. <https://doi.org/10.1016/S0190-7409(98)00006-1> | Insufficient information |
| Cadmus-Romm, D. (2004). *Emotional abuse and neglect in adolescence as predictors of psychosocial adjustment in young adulthood—ProQuest*. <https://www.proquest.com/openview/bc9deb206e763f1295cf3dad9f3f7d7a/1?pq-origsite=gscholar&cbl=18750&diss=y> | Correlation coefficients |
| Chen, W.-Y., Corvo, K., Lee, Y., & Hahm, H. C. (2017). Longitudinal Trajectory of Adolescent Exposure to Community Violence and Depressive Symptoms Among Adolescents and Young Adults: Understanding the Effect of Mental Health Service Usage. *Community Mental Health Journal*, *53*(1), 39–52. <https://doi.org/10.1007/s10597-016-0031-5> | Insufficient information |
| Choe, D. E., Stoddard, S. A., & Zimmerman, M. A. (2014). Developmental trajectories of African American adolescents’ family conflict: Differences in mental health problems in young adulthood. *Developmental Psychology*, *50*(4), 1226–1232. <https://doi.org/10.1037/a0035199> | Correlation coefficients |
| Dubowitz, H., Roesch, S., Lewis, T., Thompson, R., English, D., & Kotch, J. B. (2022). Neglect in Childhood, Problem Behavior in Adulthood. *Journal of Interpersonal Violence*, *37*(23–24), NP22047–NP22065. <https://doi.org/10.1177/08862605211067008> | Not comparable – results not pertaining to individual ACEs but classes defined by profiles of ACEs |
| Eitle, D. (2022). Examining Direct and Indirect Pathways between Adolescent Extra-Familial Criminal Victimization and Adult Mental Health. *American Journal of Criminal Justice*, *47*(5), 941–959. <https://doi.org/10.1007/s12103-021-09615-y> | Unstandardised coefficient |
| Gondek, D., Patalay, P., & Lacey, R. E. (2021). Adverse childhood experiences and multiple mental health outcomes through adulthood: A prospective birth cohort study. *SSM - Mental Health*, *1*, 100013. <https://doi.org/10.1016/j.ssmmh.2021.100013> | Insufficient information for individual ACEs |
| Heinze, J. E., Cook, S. H., Wood, E. P., Dumadag, A. C., & Zimmerman, M. A. (2018). Friendship Attachment Style Moderates the Effect of Adolescent Exposure to Violence on Emerging Adult Depression and Anxiety Trajectories. *Journal of Youth and Adolescence*, *47*(1), 177–193. <https://doi.org/10.1007/s10964-017-0729-x> | Means and standard deviations/ Point biserial correlations |
| Herrenkohl, T. I., Kosterman, R., Hawkins, J. D., & Mason, W. A. (2009). Effects of Growth in Family Conflict in Adolescence on Adult Depressive Symptoms: Mediating and Moderating Effects of Stress and School Bonding. *Journal of Adolescent Health*, *44*(2), 146–152. <https://doi.org/10.1016/j.jadohealth.2008.07.005> | Correlation coefficients |
| Hjern, A., Palacios, J., & Vinnerljung, B. (2023). Early childhood adversity and non-affective psychosis: A study of refugees and international adoptees in Sweden. *Psychological Medicine*, *53*(5), 1914–1923. <https://doi.org/10.1017/S003329172100355X> | Not comparable – hazard ratios |
| Hoprekstad, Ø. L., Hetland, J., Wold, B., Torp, H., & Valvatne Einarsen, S. (2021). Exposure to Bullying Behaviors at Work and Depressive Tendencies: The Moderating Role of Victimization From Bullying During Adolescence. *Journal of Interpersonal Violence*, *36*(23–24), 11576–11601. <https://doi.org/10.1177/0886260519900272> | Correlation coefficients |
| Horan, J. M., & Widom, C. S. (2015). Does Age of Onset of Risk Behaviors Mediate the Relationship Between Child Abuse and Neglect and Outcomes in Middle Adulthood? *Journal of Youth and Adolescence*, *44*(3), 670–682. <https://doi.org/10.1007/s10964-014-0161-4> | Means and standard deviations |
| Huurre, T., Junkkari, H., & Aro, H. (2006). Long–term Psychosocial effects of parental divorce. *European Archives of Psychiatry and Clinical Neuroscience*, *256*(4), 256–263. <https://doi.org/10.1007/s00406-006-0641-y> | Means and standard deviations |
| Iob, E., Baldwin, J. R., Plomin, R., & Steptoe, A. (2021). Adverse childhood experiences, daytime salivary cortisol, and depressive symptoms in early adulthood: A longitudinal genetically informed twin study. *Translational Psychiatry*, *11*(1), 1–10. <https://doi.org/10.1038/s41398-021-01538-w> | Insufficient information as to whether beta values are raw or standardised |
| Koenen, K. C., Moffitt, T. E., Poulton, R., Martin, J., & Caspi, A. (2007). Early childhood factors associated with the development of post-traumatic stress disorder: Results from a longitudinal birth cohort. *Psychological Medicine*, *37*(2), 181–192. <https://doi.org/10.1017/S0033291706009019> | Insufficient information - standardised solution for the path model not reported |
| Lee, C., Cronley, C., White, H. R., Mun, E.-Y., Stouthamer-Loeber, M., & Loeber, R. (2012). Racial Differences in the Consequences of Childhood Maltreatment for Adolescent and Young Adult Depression, Heavy Drinking, and Violence. *Journal of Adolescent Health*, *50*(5), 443–449. <https://doi.org/10.1016/j.jadohealth.2011.09.014> | T-test p-value and sample size |
| Moffitt, T. E., Caspi, A., Harrington, H., Milne, B. J., Melchior, M., Goldberg, D., & Poulton, R. (2007). Generalized anxiety disorder and depression: Childhood risk factors in a birth cohort followed to age 32. *Psychological Medicine*, *37*(3), 441–452. <https://doi.org/10.1017/S0033291706009640> | T-test p-value and sample size |
| Moretti, M. M., & Craig, S. G. (2013). Maternal versus paternal physical and emotional abuse, affect regulation and risk for depression from adolescence to early adulthood. *Child Abuse & Neglect*, *37*(1), 4–13. <https://doi.org/10.1016/j.chiabu.2012.09.015> | Correlation coefficients |
| Palosaari, U., Aro, H., & Laippala, P. (1996). Parental divorce and depression in young adulthood: Adolescents’ closeness to parents and self-esteem as mediating factor. *Acta Psychiatrica Scandinavica*, *93*(1), 20–26. <https://doi.org/10.1111/j.1600-0447.1996.tb10614.x> | Insufficient information |
| Ramirez, D., & Haas, S. A. (2021). The Long Arm of Conflict: How Timing Shapes the Impact of Childhood Exposure to War. *Demography*, *58*(3), 951–974. <https://doi.org/10.1215/00703370-9114715> | Unstandardised coefficient |
| Richter, L. M., Mathews, S., Nonterah, E., & Masilela, L. (2018). A longitudinal perspective on boys as victims of childhood sexual abuse in South Africa: Consequences for adult mental health. *Child Abuse & Neglect*, *84*, 1–10. <https://doi.org/10.1016/j.chiabu.2018.07.016> | Insufficient information |
| Størksen, I., Røysamb, E., Moum, T., & Tambs, K. (2005). Adolescents with a childhood experience of parental divorce: A longitudinal study of mental health and adjustment. *Journal of Adolescence*, *28*(6), 725–739. <https://doi.org/10.1016/j.adolescence.2005.01.001> | Means and standard deviations |
| van Dulmen, M. H. M., Klipfel, K. M., Mata, A. D., Schinka, K. C., Claxton, S. E., Swahn, M. H., & Bossarte, R. M. (2012). Cross-Lagged Effects Between Intimate Partner Violence Victimization and Suicidality From Adolescence Into Adulthood. *Journal of Adolescent Health*, *51*(5), 510–516. <https://doi.org/10.1016/j.jadohealth.2012.02.015> | Correlation coefficients |
| Vassallo, S., Edwards, B., Renda, J., & Olsson, C. A. (2014). Bullying in Early Adolescence and Antisocial Behavior and Depression Six Years Later: What Are the Protective Factors? *Journal of School Violence*, *13*(1), 100–124. <https://doi.org/10.1080/15388220.2013.840643> | Insufficient information |
| Wong, W. H., Kuo, W.-H., Sobolewski, C., Bhatia, I., & Ip, P. (2020). The Association Between Child Abuse and Attempted Suicide: A Retrospective Cohort Study. *Crisis*, *41*(3), 196–204. <https://doi.org/10.1027/0227-5910/a000625> | Not comparable –hazard ratios |
| Zhu, X., Griffiths, H., Eisner, M., Hepp, U., Ribeaud, D., & Murray, A. L. (2022a). Developmental associations between bullying victimization and suicidal ideation and direct self-injurious behavior in adolescence and emerging adulthood. *Journal of Child Psychology and Psychiatry*, *63*(7), 820–828. <https://doi.org/10.1111/jcpp.13529> | Not comparable – observed beta values have different interpretation from typical beta coefficients |
| Zhu, X., Griffiths, H., Eisner, M., Hepp, U., Ribeaud, D., & Murray, A. L. (2022b). Developmental Relations Between Bullying Victimization and Suicidal Ideation in Middle Adolescence and Emerging Adulthood: Do Internalizing Problems and Substance Use Mediate Their Links? *Journal of Youth and Adolescence*, *51*(9), 1745–1759. <https://doi.org/10.1007/s10964-022-01630-4> | Not comparable – as above  Nnn |

**Appendix H**

**Detailed Descriptions of how Moderators were Grouped for each Model**

Year of Publication remained as a continuous variable.

Aligning with the way a vast body of literature has conceptualized and categorized ACEs, we ran models exploring the moderating effects of ACE types grouped into the main groups: Child Abuse and Neglect vs other ACEs, Household Dysfunction vs other ACEs (Felitti et al., 1997; Cavanaugh, 2016), and aligning with more recent literature that has acknowledged bullying victimization as an ACE (Arseneault, 2018; Vaswani, 2019), bully victim vs other ACEs.

We also wanted to explore the effect of more specific ACE types using “Parental Separation” as the reference category. This was chosen as the reference as research suggests ACEs in the household dysfunction category may have less severe impacts on mental ill-health compared to ACEs where there is a maltreatment component such as bullying or child abuse (Negriff, 2021). Furthermore, studies have suggested the long-term mental health consequences associated with household-related childhood adversity can be fully mediated by the presence of childhood maltreatment (Clemens et al., 2019).

We also explored grouping of ACEs into broader umbrella categories where “Household Dysfunction” was used as the reference category which included the experiences such as household or parental criminality, household or parental morbidity, household or parental conflict, household or parental substance (mis)use, household or parental mental illness, household or parental loss, parental separation, and being in care.

ACE Count was categorized by whether a single or multiple ACE effect was measured (e.g., “more than one form of substantiated maltreatment” (multiple ACE) versus “emotional abuse” (single ACE)).

Location was categorized by continent with Europe as the reference category.

“Study Design” was categorized into “longitudinal cohorts”, where primary data were collected at both child and adult timepoints by the study team, and “data-linked studies”, where at least one wave comprised of data from a separate database or register that had been linked.

“Source of Reporting” was categorized by who provided information on ACE exposure with “Official Records” as the reference and included the other categories “Self-and/or informant” and “Self and Official”.

“Umbrella Source of Reporting” used broader categorized based on who provided information on ACE exposure with “Official Records” as the reference category and also included the categories, “Self and/or Informant”, and a category including “Self and/or Informant and Official Records”.

Cohorts used were too heterogenous to provide meaningful results and thus were not included as a moderator.

**Appendix I**

**Results of Overall Univariate Model Effects for 10 Moderator Analyses per Mental Health Outcome.**

| Mental Health Outcome | Moderator Variables | ki | *N* ES | QE (df) | *F* (df1, df2) ^a^ | p ^b^ |
| --- | --- | --- | --- | --- | --- | --- |
| Depression | Year | 28 | 97 | 281.34 (95) *** | .45 (1, 95) | .5061 |
|  | ACE Type | 28 | 97 | 165.33 (81) *** | **4.13 (15, 81) ***** | <.0001 |
|  | Umbrella ACE Type | 28 | 97 | 278.72 (90) *** | **3.54 (6, 90) **** | .0034 |
|  | CAN vs Other ACEs | 28 | 97 | 394.90 (95) *** | .49 (1, 95) | .4837 |
|  | Bully Victimisation vs Other ACEs | 28 | 97 | 373.18 (95) *** | .78 (1, 95) | .3786 |
|  | Household Dysfunction vs Other ACEs | 28 | 97 | 386.96 (95) *** | **5.00 (1, 95)*** | .0277 |
|  | ACE Count (Single vs Multiple ACEs) | 28 | 97 | 387.49 (95) *** | .12 (1, 95) | .7331 |
|  | Location | 28 | 97 | 340.91 (92) *** | 1.88 (4, 92) | .1213 |
|  | ACE Source Reporting | 28 | 97 | 301.83 (91) *** | 1.38 (5, 91) | .2406 |
|  | Study Design | 28 | 97 | 365.98 (95) *** | .42 (1, 95) | .5203 |
| Anxiety | Year | 14 | 61 | 238.90 (59) *** | 2.11 (1, 59) | .1518 |
|  | ACE Type | 14 | 61 | 259.42 (47) *** | 1.06 (13, 47) | .4147 |
|  | Umbrella ACE Type | 14 | 61 | 270.81 (54) *** | 1.21 (6, 54) | .3142 |
|  | CAN vs Other ACEs | 14 | 61 | 314.45 (59) *** | .05 (1, 59) | .8226 |
|  | Bully Victimisation vs Other ACEs | 14 | 61 | 329.38 (59) *** | .32 (1, 59) | .5721 |
|  | Household Dysfunction vs Other ACEs | 14 | 61 | 305.28 (59) *** | .006 (1, 59) | .9381 |
|  | ACE Count (Single vs Multiple ACEs) | 14 | 61 | 334.77 (59) *** | 1.58 (1, 59) | .2140 |
|  | Location | 14 | 61 | 132.17 (57) *** | **3.75 (3, 57) *** | .0158 |
|  | ACE Source Reporting | 14 | 61 | 172.10 (56) *** | .68 (4, 56) | .6120 |
|  | Study Design | 14 | 61 | 149.54 (55) *** | 1.26 (5, 550 | .2959 |
| Suicide | Year | 7 | 22 | 35.66 (20) * | .32 (1, 20) | .5797 |
|  | ACE Type | 7 | 22 | 19.39 (15) | **3.32 (6, 15) *** | .0277 |
|  | Umbrella ACE Type | 7 | 22 | 23.11 (17) | **4.02 (4, 17) *** | .0179 |
|  | CAN vs Other ACEs | 7 | 22 | 28.46 (20) | **10.61 (1, 20) **** | .0039 |
|  | Bully Victimisation vs Other ACEs | 7 | 22 | 31.99 (20) * | 3.53 (1, 20) | .0751 |
|  | Household Dysfunction vs Other ACEs | 7 | 22 | 35.21 (20) * | 1.39 (1, 20) | .2531 |
|  | ACE Count (Single vs Multiple ACEs) | 7 | 22 | 36.18 (20) * | 2.82 (1, 20) | .1085 |
|  | Location | 7 | 22 | 35.03 (20) * | .74 (1, 20) | .4001 |
|  | ACE Source Reporting | 7 | 22 | 31.88 (18) * | .81 (3, 18) | .5068 |
|  | Study Design | 7 | 22 | 34.55 (19) * | .40 (2, 19) | .6779 |
| Psychosis | Year | 5 | 17 | 17.91 (15) | 2.25(1, 15) | .1547 |
|  | ACE Type | 5 | 17 | 16.72 (13) | 1.14 (3, 13) | .3706 |
|  | Umbrella ACE Type | 5 | 17 | 17.27 (14) | 1.48 (2, 14) | .2620 |
|  | CAN vs Other ACEs | 5 | 17 | 17.90 (15) | 2.25 (1, 15) | .1514 |
|  | Bully Victimisation vs Other ACEs | 5 | 17 | 19.65 (15) | .55 (1, 15) | .4713 |
|  | Household Dysfunction vs Other ACEs | 5 | 17 | 20.01 (15) | .86 (1, 15) | .3679 |
|  | ACE Count (Single vs Multiple ACEs) | 5 | 17 | - | - | - |
|  | Location | 5 | 17 | 20.15 (15) | .69 (1, 15) | .4197 |
|  | ACE Source Reporting | 5 | 17 | - | - | - |
|  | Study Design | 5 | 17 | 17.8 (15) | 2.36 (1, 15) | .1453 |
| PTSD | Year | 4 | 14 | 21.12 (12) | 2.13 (1, 12) | .1698 |
|  | ACE Type | 4 | 14 | 13.18 (8) | .57 (5, 8) | .7248 |
|  | Umbrella ACE Type | 4 | 14 | 19.96 (12) | .10 (1, 12) | .7558 |
|  | CAN vs Other ACEs | 4 | 14 | - | - | - |
|  | Bully Victimisation vs Other ACEs | 4 | 14 | - | - | - |
|  | Household Dysfunction vs Other ACEs | 4 | 14 | - | - | - |
|  | ACE Count (Single vs Multiple ACEs) | 4 | 14 | 19.96 (12) | .10 (1, 12) | .7558 |
|  | Location | 4 | 14 | 10.04 (12) | 13.25 (1, 12) | .0034 |
|  | ACE Source Reporting | 4 | 14 | - | - | - |
|  | Study Design | 4 | 14 | - | - | - |

Note: * =p<.05, ** = p<.005, *** = p<.0001, ki = number of independent studies, N ES = number of effect sizes. Reference category ACE Type = parental; separation, reference category Umbrella ACE Type = Household Dysfunction. Missing data represents analyses that were unable to be conducted due to all data being homogeneous (e.g., all ACEs being single ACEs with no multiple ACE comparisons for the psychosis model).

a Omnibus test

b p value of omnibus test

**Appendix J**

**Full moderator analysis results for Depression**

Table of results showing moderating effects of each ACE Type for Depression Model

| ACE Type | estimate | intercept + estimate | se | t (df) | p | Lower CI | Upper CI |
| --- | --- | --- | --- | --- | --- | --- | --- |
| intercept (parental separation) | 1.31 | - | .13 | 10.40 (81) *** | <.0001 | 1.06 | 1.56 |
| Bully victimisation | .42 | 1.73 | .21 | 1.97 (81) | .0526 | -.005 | .84 |
| Both bully victim and perp | .92 | 2.23 | .42 | 2.19 (81) * | .0311 | .09 | 1.76 |
| Both bully victim and maltreatment | 1.66 | 2.97 | .62 | 2.67 (81) ** | .0092 | .42 | 2.89 |
| CAN | .44 | 1.75 | .14 | 3.06 (81) ** | .0030 | .15 | .72 |
| Multi-type CAN | .01 | 1.32 | .17 | .03 (81) | .9735 | -.34 | .35 |
| Cumulative ACEs | .51 | 1.82 | .21 | 2.45 (81) * | .0163 | .10 | .92 |
| HH/Parental Conflict | .64 | 1.95 | .22 | 2.91 (81) ** | .0046 | .20 | 1.08 |
| HH/Parental Criminality | .13 | 1.44 | .22 | .60 (81) | .5504 | -.31 | .57 |
| HH/Parental Loss | .03 | 1.34 | .15 | .19 (81) | 8464 | -.27 | .33 |
| HH/Parental Substance (mis)use | -.05 | 1.26 | .19 | -.25 (81) | .8058 | -.44 | .34 |
| HH/Parental Morbidity | .05 | 1.36 | .17 | .28 (81) | .7837 | -.29 | .38 |
| In care | .40 | 1.35 | .43 | .93 (81) | .3556 | -.45 | 1.25 |
| Violence exposure | -.04 | 1.27 | .15 | -.27 (81) | .7872 | -.34 | .26 |
| Loss or Violence Exposure | .84 | 2.15 | .61 | 1.37 (81) | .1733 | -.38 | 2.06 |
| Parental mental illness | -.19 | 1.12 | .17 | -1.1 (81) | .2756 | -.53 | .15 |

Table of results showing moderating effects of each Umbrella ACE Type for Depression Model

| Umbrella ACE Type | estimate | estimate + intercept | se | t (df) | p | Lower CI | Upper CI |
| --- | --- | --- | --- | --- | --- | --- | --- |
| intercept (Household Dysfunction) | 1.38 | - | .11 | 12.93 (90) *** | <.0001 | 1.17 | 1.59 |
| Bully victimisation | .47 | 1.85 | .23 | 2.03 (90) * | .0448 | .01 | .93 |
| Both bully victim and maltreatment | 1.59 | 2.97 | .64 | 2.49 (90) * | .0145 | .32 | 2.90 |
| Any CAN | 1.31 | 1.69 | .11 | 2.87 (90) ** | .0051 | .09 | .52 |
| Cumulative ACEs | .48 | 1.86 | .22 | 2.19 (90) * | .0308 | .05 | .91 |
| Violence exposure | -.07 | 1.31 | .15 | -.49 (90) | .6280 | -.37 | .22 |
| Loss or Violence Exposure | .93 | 2.31 | .60 | 1.54 (90) | .1269 | -.27 | 2.12 |

Table of results showing moderating effects of Year of Publication for Depression Model

| Year of Publication | estimate | estimate + intercept | se | t (df) | p | Lower CI | Upper CI |
| --- | --- | --- | --- | --- | --- | --- | --- |
| intercept | 21.92 | - | 30.44 | .72 (95) | .4731 | -38.51 | 82.35 |
| Year | -.01 | 21.91 | .02 | -.67 (95) | .5061 | -0.04 | .02 |

Table of results showing moderating effects of ACE Count for Depression Model

| ACE Count | estimate | estimate + intercept | se | t (df) | p | Lower CI | Upper CI |
| --- | --- | --- | --- | --- | --- | --- | --- |
| intercept (single) | 1.60 | - | .08 | 19.13 (95) *** | <.0001 | 1.43 | 1.77 |
| Multiple | .05 | 1.65 | .15 | .34 (95) | .7331 | -.25 | .35 |

Table of results showing moderating effects of Location for Depression Model

| Location | estimate | estimate + intercept | se | t (df) | p | Lower CI | Upper CI |
| --- | --- | --- | --- | --- | --- | --- | --- |
| intercept (North America) | 1.60 | - | .11 | 14.78 (92) *** | <.0001 | 1.39 | 1.82 |
| Africa | -.40 | 1.2 | .31 | -1.31 (92) | .1927 | -1.02 | .21 |
| Australia/ Oceania | .18 | 1.78 | .29 | .63 (92) | .5329 | -.40 | .77 |
| Europe | -.06 | 1.54 | .17 | -.34 (92) | .7369 | -.38 | .27 |
| South America | .80 | 2.4 | .38 | 2.12 (92) * | .0368 | .05 | 1.56 |

Table of results showing moderating effects of Source of Reporting for Depression Model

| Source of Reporting | estimate | estimate + intercept | se | t (df) | p | Lower CI | Upper CI |
| --- | --- | --- | --- | --- | --- | --- | --- |
| intercept (Official Records) | 1.69 | - | .19 | 8.71 (94) *** | <.0001 | 1.30 | 2.07 |
| Self and Official | 0.73 | 1.76 | .39 | .18 (94) | .8537 | -.71 | .85 |
| Self (including informant) | -.11 | 1.58 | .22 | -.53 (94) | .5987 | -.54 | .32 |

Table of results showing moderating effects of Study Design for Depression Model

| Study Design | estimate | estimate + intercept | se | t (df) | p | Lower CI | Upper CI |
| --- | --- | --- | --- | --- | --- | --- | --- |
| intercept (Data-linked) | 1.71 | - | .19 | 9.19 (95) *** | <.0001 | 1.34 | 2.08 |
| Longitudinal | -.13 | 1.58 | .21 | -.65 (95) | .5203 | -.55 | .28 |

Table of results showing moderating effects of Household Dysfunction compared to other ACE Types for Depression Model

| ACE Type | estimate | estimate + intercept | se | t (df) | p | Lower CI | Upper CI |
| --- | --- | --- | --- | --- | --- | --- | --- |
| intercept | 1.68 | - | .09 | 18.73 (95)*** | <.0001 | 1.50 | 1.85 |
| Household Dysfunction vs All Other ACE Types | -.23 | 1.45 | .10 | -2.24 (95)* | .0277 | -.43 | -.03 |

Table of results showing moderating effects of Child Abuse and Neglect compared to other ACE Types for Depression Model

| ACE Type | estimate | estimate + intercept | se | t (df) | p | Lower CI | Upper CI |
| --- | --- | --- | --- | --- | --- | --- | --- |
| intercept | 1.57 | - | .09 | 16.57 (95) *** | <.0001 | 1.38 | 1.76 |
| CAN vs All Other ACE Types | .08 | 1.65 | .11 | .70 (95) | .4837 | -.14 | .29 |

Table of results showing moderating effects of Bullying compared to other ACE Types for Depression Model

| ACE Type | estimate | estimate + intercept | se | t (df) | p | Lower CI | Upper CI |
| --- | --- | --- | --- | --- | --- | --- | --- |
| intercept | 1.58 | - | .09 | 18.24 (95) *** | <.0001 | 1.41 | 1.75 |
| Bullying vs All Other ACE Types | .20 | 1.78 | .22 | .88 (95) | .3786 | -.25 | .64 |

**Appendix K**

**Full moderator analysis results for Anxiety**

*Note: There is no table of results showing moderating effects of each ACE Type for Anxiety Model as data was too heterogenous to be divided into specific ACEs*

Table of results showing moderating effects of each Umbrella ACE Type for Anxiety Model

| Umbrella ACE Type | estimate | estimate + intercept | se | t (df) | p | Lower CI | Upper CI |
| --- | --- | --- | --- | --- | --- | --- | --- |
| intercept (Household Dysfunction) | 1.69 | - | .21 | 7.93 (55) *** | <.0001 | 1.26 | 2.12 |
| Bully victimisation | .49 | 2.18 | .33 | 1.46 (55) | .1489 | -.18 | 1.15 |
| Both bully victim and maltreatment | -.48 | 1.21 | .42 | -1.14 (55) | .2588 | -1.33 | .37 |
| Any CAN | .06 | 1.75 | .17 | .34 (55) | .7380 | -.29 | .41 |
| Cumulative ACEs | -.19 | 1.5 | .23 | -.85 (55) | .4008 | -.65 | .26 |
| Violence exposure | .03 | 1.66 | .22 | .15 (55) | .8790 | -.41 | .47 |

Table of results showing moderating effects of Year of Publication for Anxiety Model

| Year of Publication | estimate | estimate + intercept | se | t (df) | p | Lower CI | Upper CI |
| --- | --- | --- | --- | --- | --- | --- | --- |
| intercept | 107.61 | - | 72.89 | 1.48 (59) | .1452 | -38.24 | 253.45 |
| Year | -.05 | 107.56 | .04 | -1.45 (59) | .1518 | -.12 | .02 |

Table of results showing moderating effects of ACE Count for Anxiety Model

| ACE Count | estimate | estimate + intercept | se | t (df) | p | Lower CI | Upper CI |
| --- | --- | --- | --- | --- | --- | --- | --- |
| intercept (single) | 1.82 | - | .17 | 10.84 (59) *** | <.0001 | 1.49 | 2.16 |
| Multiple | -.19 | 1.63 | .15 | -1.26 (59) | .2140 | -.50 | .12 |

Table of results showing moderating effects of Location for Anxiety Model

| Location | estimate | estimate + intercept | se | t (df) | p | Lower CI | Upper CI |
| --- | --- | --- | --- | --- | --- | --- | --- |
| intercept (North America) | 1.81 | - | .18 | 10.28 (57) *** | <.0001 | 1.46 | 2.16 |
| Africa | -.86 | .95 | .36 | -2.35 (57) * | .022 | -1.58 | -.13 |
| Australia/ Oceania | .57 | 2.38 | .35 | 1.62 (57) | .1107 | -.13 | 1.26 |
| Europe | -.21 | 1.6 | .25 | -.86 (57) | .3942 | -.71 | .28 |

Table of results showing moderating effects of Source of Reporting for Anxiety Model

| Source of Reporting | estimate | estimate + intercept | se | t (df) | p | Lower CI | Upper CI |
| --- | --- | --- | --- | --- | --- | --- | --- |
| intercept (Official Records) | 2.11 | - | .32 | 6.16 (58) *** | <.0001 | 1.42 | 2.79 |
| Self and Official | 1.29 | 3.4 | 1.41 | .91 (58) | .3660 | -1.54 | 4.12 |
| Self (including informant) | -.45 | 1.66 | .39 | -1.18 (58) | .2433 | -1.22 | .32 |

Table of results showing moderating effects of Study Design for Anxiety Model

| Study Design | estimate | estimate + intercept | se | t (df) | p | Lower CI | Upper CI |
| --- | --- | --- | --- | --- | --- | --- | --- |
| intercept (Data-linked) | 2.18 | - | .33 | 6.58 (59) *** | <.0001 | 1.52 | 2.85 |
| Longitudinal | -.53 | 1.65 | .38 | -1.41 (59) | .1644 | -1.28 | .22 |

Table of results showing moderating effects of Household Dysfunction compared to other ACE Types for Anxiety Model

| ACE Type | estimate | estimate + intercept | se | t (df) | p | Lower CI | Upper CI |
| --- | --- | --- | --- | --- | --- | --- | --- |
| intercept | 1.79 | - | .17 | 10.47 (59) *** | <.0001 | 1.45 | 2.13 |
| Household Dysfunction vs All other ACE Types | -.01 | 1.78 | .14 | -.08 (59) | .9381 | -.30 | .28 |

Table of results showing moderating effects of Child Abuse and Neglect compared to other ACE Types for Anxiety Model

| ACE Type | estimate | estimate + intercept | se | t (df) | p | Lower CI | Upper CI |
| --- | --- | --- | --- | --- | --- | --- | --- |
| intercept | 1.80 | - | .18 | 10.17 (59) *** | <.0001 | 1.45 | 2.15 |
| CAN vs All other ACE Types | -.03 | 1.77 | .13 | -.23 (59) | .8226 | -.30 | .24 |

Table of results showing moderating effects of Bullying compared to other ACE Types for Anxiety Model

| ACE Type | estimate | estimate + intercept | se | t (df) | p | Lower CI | Upper CI |
| --- | --- | --- | --- | --- | --- | --- | --- |
| intercept | 1.75 | - | .19 | 9.38 (59) *** | <.0001 | 1.37 | 2.12 |
| Bullying vs All other ACE Types | .16 | 1.59 | .28 | .57 (59) | .5721 | -.40 | .72 |

**Appendix L**

**Full moderator analysis results for Suicide**

*Note: There is no table of results showing moderating effects of each ACE Type for the Suicide Model as data was too heterogenous to be divided into specific ACEs.*

*There is no table of results showing moderating effects of Household Dysfunction compared to other ACE Types for the Suicide Model due to homogeneous data.*

*There is no table of results showing moderating effects of Study Design for the Suicide Model due to homogeneous data.*

*The reference group for Source of Reporting for the Suicide Model was different than the other mental health outcomes due to homogeneous data.*

Table of results showing moderating effects of each Umbrella ACE Type for Suicide Model

| Umbrella ACE Type | estimate | estimate + intercept | se | t (df) | p | Lower CI | Upper CI |
| --- | --- | --- | --- | --- | --- | --- | --- |
| intercept (Household Dysfunction) | 1.46 | - | .13 | 11.02 (17) *** | <.0001 | 1.18 | 1.74 |
| Bully victimisation | -.07 | 1.39 | .17 | -.40 (17) | .6929 | -.42 | .29 |
| Both bully victim and maltreatment | .55 | .91 | .42 | 1.31 (17) | .2066 | -.33 | 1.43 |
| Any CAN | -.42 | 1.04 | .29 | -1.48 (17) | .1560 | -1.03 | .18 |
| Violence exposure | -.33 | 1.13 | .14 | -2.45 (17) * | .0255 | -.62 | -.05 |

Table of results showing moderating effects of Year of Publication for Suicide Model

| Year of Publication | estimate | estimate + intercept | se | t (df) | p | Lower CI | Upper CI |
| --- | --- | --- | --- | --- | --- | --- | --- |
| intercept | -15.17 | - | 29.22 | -.52 (20) | .6094 | -76.13 | 45.79 |
| Year | .0008 | -15.17 | .01 | .56 (20) | .5797 | -.02 | .04 |

Table of results showing moderating effects of ACE Count for Suicide Model

| ACE Count | estimate | estimate + intercept | se | t (df) | p | Lower CI | Upper CI |
| --- | --- | --- | --- | --- | --- | --- | --- |
| intercept (single) | 1.26 | - | .07 | 18.99 (20) *** | <.0001 | 1.12 | 1.40 |
| Multiple | .69 | 1.95 | .41 | 1.68 (20) | .1085 | -.17 | 1.55 |

Table of results showing moderating effects of Location for Suicide Model

| Location | estimate | estimate + intercept | se | t (df) | p | Lower CI | Upper CI |
| --- | --- | --- | --- | --- | --- | --- | --- |
| intercept (North America) | 1.23 | - | .08 | 14.64 (20) *** | <.0001 | 1.06 | 1.41 |
| Europe | .12 | 1.35 | .14 | .86 (20) | .4001 | -.18 | .42 |

Table of results showing moderating effects of Source of Reporting for Suicide Model

| Source of Reporting | estimate | estimate + intercept | se | t (df) | p | Lower CI | Upper CI |
| --- | --- | --- | --- | --- | --- | --- | --- |
| intercept (Self (including informant)) | 1.29 | - | .08 | 15.37 (20) *** | <.0001 | 1.12 | 1.47 |
| Self and Official | -.02 | 1.27 | .24 | -.08 (20) | .9347 | -.52 | .48 |

Table of results showing moderating effects of Child Abuse and Neglect compared to other ACE Types for Suicide Model

| ACE Type | estimate | estimate + intercept | se | t (df) | p | Lower CI | Upper CI |
| --- | --- | --- | --- | --- | --- | --- | --- |
| intercept | 1.42 | - | .08 | 17.43 (20) *** | <.0001 | 1.25 | 1.59 |
| CAN vs All other ACE Types | -.29 | 1.13 | .09 | -3.26 (20) ** | .0039 | -.47 | -.10 |

Table of results showing moderating effects of Bullying compared to other ACE Types for Suicide Model

| ACE Type | estimate | estimate + intercept | se | t (df) | p | Lower CI | Upper CI |
| --- | --- | --- | --- | --- | --- | --- | --- |
| intercept | 1.19 | - | .09 | 13.99 (20) *** | <.0001 | 1.01 | 1.37 |
| Bullying vs All other ACE Types | .28 | 1.47 | .15 | 1.88 (15) **^.^** | .0751 | -.03 | .58 |

Table of results showing moderating effects of Household Dysfunction compared to other ACE Types for Suicide Model

| ACE Type | estimate | estimate + intercept | se | t (df) | p | Lower CI | Upper CI |
| --- | --- | --- | --- | --- | --- | --- | --- |
| intercept | 1.24 | - | .07 | 17.24 (20) *** | <.0001 | 1.09 | 1.39 |
| Household Dysfunction vs All other ACE Types | .22 | 1.46 | .19 | 1.18 (20) | .2531 | -.17 | .62 |

**Appendix M**

**Full moderator analysis results for PTSD**

*Note: There is no table of results showing moderating effects of specific ACE Type, Umbrella ACE Type, Source of Reporting, and Study Design for* PTSD *Model as data was too homogeneous.*

Table of results showing moderating effects of Year of Publication for PTSD Model

| Year of Publication | estimate | estimate + intercept | se | t (df) | p | Lower CI | Upper CI |
| --- | --- | --- | --- | --- | --- | --- | --- |
| intercept | -78.64 | - | 55.45 | -1.42 (12) | .1816 | -199.45 | 42.18 |
| Year | .04 | -78.6 | .03 | 1.46 (12) | .1698 | -.02 | .10 |

Table of results showing moderating effects of ACE Count for PTSD Model

| ACE Count | estimate | estimate + intercept | se | t (df) | p | Lower CI | Upper CI |
| --- | --- | --- | --- | --- | --- | --- | --- |
| intercept (single) | 2.28 | - | .28 | 8.29 (12) *** | <.0001 | 1.68 | 2.89 |
| Multiple | .23 | 2.51 | .73 | .32 (12) | .7558 | -1.36 | 1.83 |

Table of results showing moderating effects of Location for PTSD Model

| Location | estimate | estimate + intercept | se | t (df) | p | Lower CI | Upper CI |
| --- | --- | --- | --- | --- | --- | --- | --- |
| intercept (North America) | 1.74 | - | .13 | 12.90 (12)*** | <.0001 | 1.44 | 2.03 |
| Australia/ Oceania | 1.22 | 2.96 | .33 | 3.64 (12)** | .0034 | .49 | 1.94 |

**Appendix N**

**Full moderator analysis results for Psychosis**

*Note: There is no table of results showing moderating of each ACE Type for the Psychosis Model as there was not enough data to be divided into specific ACEs.*

*There is no table of results showing moderating effects of ACE Count for the Psychosis Model due to homogeneous data.*

*The reference group for Location for the Psychosis Model was different than the other mental health outcomes due to no data containing North America.*

Table of results showing moderating effects of each Umbrella ACE Type for Psychosis Model

| Umbrella ACE Type | estimate | estimate + intercept | se | t (df) | p | Lower CI | Upper CI |
| --- | --- | --- | --- | --- | --- | --- | --- |
| intercept (Household Dysfunction) | .71 | - | .69 | 1.03 (14) | .3190 | -.76 | 2.18 |
| Bully victimisation | .60 | 1.31 | .69 | .87 (14) | .4001 | -.88 | 2.08 |
| Any CAN | 1.23 | 1.94 | .80 | 1.53 (14) | .1485 | -.50 | 2.96 |

Table of results showing moderating effects of Year of Publication for Psychosis Model

| Year of Publication | estimate | estimate + intercept | se | t (df) | p | Lower CI | Upper CI |
| --- | --- | --- | --- | --- | --- | --- | --- |
| intercept | 212.56 | - | 140.94 | 1.51 (15) | .1523 | -87.86 | 512.97 |
| Year | -.10 | 212.46 | .07 | -1.50 (15) | .1547 | -.25 | .04 |

Table of results showing moderating effects of Location for Psychosis Model

| Location | estimate | estimate + intercept | se | t (df) | p | Lower CI | Upper CI |
| --- | --- | --- | --- | --- | --- | --- | --- |
| intercept (Europe) | .79 | - | .68 | 1.16 (15) | .2635 | -.66 | 2.23 |
| Australia/ Oceania | .57 |  | .68 | .83 (15) | .4197 | -.89 | 2.03 |

Table of results showing moderating effects of Source of Reporting for Psychosis Model

| Source of Reporting | estimate | estimate + intercept | se | t (df) | p | Lower CI | Upper CI |
| --- | --- | --- | --- | --- | --- | --- | --- |
| intercept (Official Records) | 1.94 | - | .43 | 4.47 (14) | .0005 | 1.01 | 2.87 |
| Self and Official | .31 |  | 3.12 | .10 (14) | .9223 | -6.39 | 7.01 |
| Self (including informant) | -.65 |  | .44 | -1.46 (14) | .1671 | -1.60 | .30 |

Table of results showing moderating effects of Study Design for Psychosis Model

| Study Design | estimate | estimate + intercept | se | t (df) | p | Lower CI | Upper CI |
| --- | --- | --- | --- | --- | --- | --- | --- |
| intercept (Data-linked) | 1.94 | - | .41 | 4.69 (15) | .0003 | 1.06 | 2.83 |
| Longitudinal | -.65 |  | .42 | -1.54 (15) | .1453 | -1.56 | .25 |

Table of results showing moderating effects of Household Dysfunction compared to other ACE Types for Psychosis Model

| ACE Type | estimate | estimate + intercept | se | t (df) | p | Lower CI | Upper CI |
| --- | --- | --- | --- | --- | --- | --- | --- |
| intercept | 1.36 | - | .10 | 13.74 (15) | <.0001 | 1.15 | 1.57 |
| Household Dysfunction vs All Other ACE Types | -.65 |  | .70 | -.93 (15) | .3679 | -2.13 | .84 |

Table of results showing moderating effects of Child Abuse and Neglect compared to other ACE Types for Psychosis Model

| ACE Type | estimate | estimate + intercept | se | t (df) | p | Lower CI | Upper CI |
| --- | --- | --- | --- | --- | --- | --- | --- |
| intercept | 1.29 | - | .09 | 14.20 (15) | <.0001 | 1.10 | 1.49 |
| CAN vs All Other ACE Types | .65 |  | .43 | .1.50 (15) | .1541 | -.27 | 1.56 |

Table of results showing moderating effects of Bullying compared to other ACE Types for Psychosis Model

| ACE Type | estimate | estimate + intercept | se | t (df) | p | Lower CI | Upper CI |
| --- | --- | --- | --- | --- | --- | --- | --- |
| intercept | 1.60 | - | .37 | 4.27 (15) | .0007 | .80 | 2.40 |
| Bullying vs All Other ACE Types | -.29 |  | .39 | -.74 (15) | .4713 | -1.11 | .54 |
